# Interventions to build resilience and to ameliorate negative psychosocial effects of the COVID-19 pandemic on children and adolescents: a systematic review and meta-analysis

**DOI:** 10.1007/s00787-023-02280-y

**Published:** 2023-08-13

**Authors:** Flora Wendel, Stephan Bender, Eva Breitinger, Michaela Coenen, Julia Hummel, Gisela Immich, Michaela Kirschneck, Vera Klünder, Angela M. Kunzler, Klaus Lieb, Ani Movsisyan, Lydia Y. Li, Ulrike Ravens-Sieberer, Eva Rehfuess, Stephan Voss, Caroline Jung-Sievers

**Affiliations:** 1 Institute for Medical Information Processing, Biometry and Epidemiology IBE, Faculty of Medicine, Chair of Public Health and Health Services Research, LMU Munich, Munich, Germany; 2 Pettenkofer School of Public Health, Munich, Germany; 3 Department of Child and Adolescent Psychiatry, Psychosomatics, and Psychotherapy, University of Cologne, Faculty of Medicine and University Hospital Cologne, Cologne, Germany; 4 Leibniz Institute for Resilience Research (LIR), Mainz, Germany; 5 Institute for Evidence in Medicine, Faculty of Medicine, Medical Center – University of Freiburg, University of Freiburg, Freiburg, Germany; 6 Department of Child and Adolescent Psychiatry, Psychotherapy, and Psychosomatics, Research Unit Child Public Health, University Medical Center Hamburg-Eppendorf, Hamburg, Germany

**Keywords:** Mental health, Resilience, COVID-19, Children, Adolescents

## Abstract

The psychosocial health of children and adolescents has been particularly affected by the COVID-19 pandemic. Containment measures have restricted social development, education and recreational activities, may have increased family conflicts and, in many cases, led to feelings of loneliness, sleep disturbances, symptoms of anxiety and depression. We conducted a systematic review to identify interventions that seek to ameliorate these detrimental effects of the COVID-19 pandemic and to build resilience in children and adolescents. Literature searches were conducted in the databases MEDLINE, EMBASE, PsycINFO, CENTRAL, WHO COVID-19 Global literature on coronavirus disease and Cochrane COVID-19 Study Register (up to 30 June 2022). The searches retrieved 9557 records of which we included 13 randomized-controlled trials (RCTs) for evidence synthesis. Included studies predominantly implemented online group sessions for school-aged children with either a psychological component, a physical activity component, or a combination of both. A meta-analysis of seven studies on anxiety and five on depressive symptoms provided evidence for a positive effect of interventions by reducing anxiety (Standardized Mean Difference (SMD) (95% CI): − 0.33 (− 0.59; − 0.06)) and depressive symptoms (SMD (95% CI): − 0.26 (− 0.36; − 0.16)) compared to the control interventions. Studies also showed improvements in positive mental health outcomes, such as resilience (n = 2) and mental and psychological wellbeing (n = 2). Exploratory subgroup analyses suggested a greater effectiveness of interventions that (i) are of higher frequency and duration, (ii) enable personal interaction (face-to-face or virtually), and (iii) include a physical activity component. Almost all studies were judged to be at high risk of bias and showed considerable heterogeneity. Further research may focus on the contribution of different intervention components or distinct subgroups and settings, and should examine children and adolescents over longer follow-up periods.

**Supplementary Information:**

The online version contains supplementary material available at 10.1007/s00787-023-02280-y.

## Introduction

The COVID-19 pandemic and its broader social and economic consequences continue to affect our health and wellbeing. While children and adolescents have comparatively small risks of severe illness and mortality from a SARS-CoV-2 infection, they are among the most vulnerable in terms of psychosocial consequences and have been negatively affected in several ways [1, 2]. Containment measures such as lockdowns and school closures have restricted social development, play and education. As such, schooling and leisure time activities were discontinued or converted to online formats [3]. Contact to peers, friends and family was heavily restricted and physical activity decreased for this age group in several phases of the pandemic. Negative consequences did not affect all children and adolescents equally, and the pandemic may also have led to positive developments in some individuals. On the other hand, feelings of loneliness, boredom, fear of infection, being separated from parents (due to self-isolation or quarantine), or loss of relatives have been increasingly reported by children and adolescents [1]. Overall, the COVID-19 pandemic led to a decrease in children’s and adolescents’ general wellbeing, an increase of mental health problems and psychiatric diagnoses, and a growing demand for (non-specialized and specialized) mental health care. Specifically, a rise in depressive symptoms, anxiety, suicidal ideation, loneliness, sleep disturbance, eating disorders, gaming and increased screen time has been reported [4–8].

Apart from the effects on the individual child or adolescent, the pandemic situation affected parents and whole family constructs [9], with a rise in parental stress, interparental and parent–child conflicts [10], child maltreatment [11, 12] and intimate partner violence [13].

With the COVID-19 pandemic representing a stressor at individual as well as family and societal levels, it is important to explore how mental health can be promoted, maintained, or restored during pandemic circumstances and crises, especially for the young [14]. In this context, resilience as the maintenance or quick recovery of mental health despite exposure to a stressor or adversity [14, 15] is an important concept and target for intervention. This concept encompasses i) processes of dynamically adapting to stressors and ii) promoting resilience factors. A multitude of resilience-conducive supporting factors have been discussed in the literature, such as perceived social support, coping strategies, cognitive emotion regulation strategies, hope, self-efficacy, or regulatory flexibility [14]. While these are protective factors at the individual level, the immediate (family) and wider environment (school, community) play an important role for children’s and adolescents’ resilience, too. Factors that can contribute to maintaining or regaining mental health comprise, for example, family coherence, family problem-solving, close relationships, school routines, nurturing by the school community or community action [16].

Previous evidence on mental health interventions to build resilience or prevent mental illness for children and adolescents was published before the COVID-19 pandemic [17–19] and in times of other health emergencies [20, 21], such as armed conflicts (e.g. war, terrorism) or natural disasters (e.g. earthquakes, hurricanes). However, it is unclear whether these findings can be readily applied in the current pandemic situation. While most emergency situations come along with fear, loss, potential illness and death and (short) disruptions of public services and economic activity, the COVID-19 pandemic was additionally characterized by frequent, repeated and often severe restrictions to personal contacts and social life. Such face-to-face contacts, also outside the immediate family, are particularly important for children, but were highly limited over prolonged periods of time and, at times, were not possible at all. Moreover, the pandemic affected (nearly) all children and adolescents worldwide, whereas natural catastrophes and armed conflict are usually restricted to a (small) geographical area. Similarly, findings for interventions that were studied during other epidemics (e.g. Ebola) [22, 23] may also only have limited informative value in the present pandemic context, because of differences in the characteristics of the pathogen and its harmfulness, in the spread of disease, and in containment measures and their duration [23–25].

A previous systematic review conducted by Boldt et al. at an early stage of the COVID-19 pandemic [26] identified multiple study protocols of interventions aiming to mitigate the negative psychosocial consequences of the COVID-19 pandemic on children and adolescents. However, at the time of the search (up to 25 September 2020), several randomized-controlled trials had been initiated but none had been completed.

The objective of this systematic review was therefore to assess the effectiveness of interventions that seek to build resilience and to ameliorate the psychosocial effects of the COVID-19 pandemic on children and adolescents, updating the previous systematic review by Boldt et al. and informing pandemic preparedness.

## Methods

We conducted this systematic review largely following the methodological standards and quality criteria laid out in the Cochrane handbook [27]. Reporting is aligned with the PRISMA reporting guidelines for systematic reviews and meta-analyses [28].

Building on the previous systematic review by Boldt et al. [26], we extended and adapted the previous study protocol for this review. The protocol was registered a priori in the Open Science Framework (https://osf.io) [29].

### Inclusion and exclusion criteria

Inclusion and exclusion criteria are based on the PICOS scheme, defining specific criteria for populations, interventions, controls, outcomes and study designs. Regarding the population, studies were eligible for inclusion if they targeted either children and adolescents up to the age of 19 [30] and/or their parents or primary caregivers. Participants with elevated vulnerability, depressive symptoms or anxiety scores, were included, provided that they had no specific psychiatric diagnoses or chronic conditions or were currently undergoing treatment, such as psychotherapy or psychological counseling, formal psychosocial services or mental health support, or pharmacological treatment.

Interventions had to aim at mitigating psychosocial consequences of the pandemic or at building resilience among the population of interest. We did not set restrictions in terms of modes of delivery (e.g. individual, group, online, in-person, self-guided etc.) or different theoretical foundations (e.g. cognitive behavioral, social learning, etc.) or components of interventions (e.g. psychoeducation, structured social, recreational or sportive activities, psychological counseling, family support, etc.).

We included studies with any of the following control conditions: No treatment, waitlist-control, active control (e.g. health education) or alternative active control (intervention lacking the essential component, e.g. mandala drawing).

Outcome categories were chosen based on those identified as important for decision-makers as part of the ongoing work to develop a World Health Organization (WHO) guideline on parenting programs to prevent child maltreatment and promote positive development [31]. Additional outcome categories relevant to the review question were added based on expert opinion. Included studies had to report on outcomes such as child/adolescent externalizing problems (e.g. conduct problems, oppositional behavior, delinquency, drug or alcohol use, suicidal or self-harm behavior, eating disorder), internalizing problems (e.g. anxiety, depression, posttraumatic stress disorder (PTSD), psychosocial distress, perceived stress, suicidal thoughts, sleep disturbance, eating disorder), child/adolescent maltreatment, resilience, self-efficacy, mental wellbeing or quality of life. Outcomes of interest at the parents or caregiver level comprised resilience, parental stress, and parenting skills and behaviors.

Regarding study design, inclusion of studies was restricted to randomized-controlled trials (RCT) and cluster-randomized-controlled trials (cRCTs). Further, we included protocols of RCTs and cRCTs, and reported them separately (in the Appendix). As the context of interest was the COVID-19 pandemic, only studies conducted after 1 January 2020 up to 30 June 2022 with a focus on the COVID-19 pandemic and its consequences were included.

We excluded studies that focused on participants with pre-existing psychiatric or chronic somatic conditions, as the aim was to study the general population with a focus on prevention and mental health promotion. Participants admitted to hospital (isolation) wards were likewise excluded. Non-pharmacological interventions (also called public health and social measures), as well as interventions with pharmacological components were excluded. All studies that solely reported on somatic outcomes or any other outcomes not listed above were excluded.

### Information sources and search strategy

We conducted systematic literature searches in MEDLINE (via Ovid), EMBASE (via Ovid), CENTRAL and PsycINFO (via EBSCO) up until 30 June 2022.

We used a combination of terms relating to the COVID-19 pandemic (e.g. “COVID-19”, “SARS-CoV2”), population (e.g. “children”, “adolescents”, “parents”, “families”, “caregivers”), interventions (e.g. “support”, “counselling”, “activities”, “psychosocial”, “psychological”), and psychosocial outcomes (e.g. “behavioral problems”, “distress”, “anxiety”, “depression”, “resilience”, “child maltreatment”). The full search strategy for MEDLINE and other databases can be found in Appendix A. We additionally searched the COVID-19-specific databases “WHO COVID-19 Global literature on coronavirus disease” and “Cochrane COVID-19 Study Register”. Literature search and title and abstract screening were performed in English only. Full-text screening and study inclusion were limited to studies published in Chinese, English, German, Italian, Russian, Spanish, and French as these languages were covered by the review team. Studies published ahead of print were also considered. Additionally, we screened the reference lists of relevant review articles for identification of further relevant studies.

### Selection process

After removal of duplicate studies and a calibration assessment of 30 studies, all records were screened on a title and abstract basis by a single review author (EB, GI, JH, LY, MK, SV, FW, CJS). Twenty percent of all titles and abstracts were independently screened by a second review author (EB, GI, JH, LY, MK, SV, FW). Given the very high degree of agreement between review authors, the team decided that double-screening of all titles and abstracts was not necessary. Generally, a conservative approach was taken where records with uncertainties and no clear exclusion criteria were moved to the full-text stage of the screening process. In the next step, two review authors independently assessed the full texts of all studies (EB, GI, JH, LY, MK, SV, FW). Uncertainties were resolved through discussion and by consulting a third review author when necessary.

EndNote [32] was used to store and de-duplicate studies. For the screening of titles and abstracts, we used Rayyan, a web-based application for facilitating citation screening for systematic reviews [33]. At the full-text screening stage, we documented the reasons for exclusion using Microsoft Excel spreadsheets [34]. Reasons of exclusion were documented hierarchically, meaning that in case of multiple reasons for exclusion, only the first reason (study design > COVID-19 pandemic > population > intervention > outcome) was documented. The list of all studies screened at this stage with the respective reasons for exclusion is provided in Appendix B, Table A1.

### Data extraction process

Study characteristics and study data were extracted onto an a priori developed Microsoft Excel sheet by one review author (LY, JH, SV, FW, CJS) [34]. All extracted study data was checked for completeness and correctness by a second review author (LY, JH, SV, FW).

Extracted data items comprised information on (i) the study (e.g. publication date, study design, country in which the study was conducted, information on recruitment, inclusion criteria, characteristics and number of participants), (ii) interventions and control conditions (e.g. mode of delivery, theory, frequency and intensity), and (iii) outcomes (e.g. outcome measurement, effect sizes, follow-up dates, statistical methods, additional analyses). The full list of extracted data items can be found in Appendix C.

### Risk of bias assessment

To assess the risk of bias of the included studies, we used the Cochrane Risk of Bias tool 2 (RoB2) [35], which is the recommended tool for critically appraising RCTs. We assessed the overall risk, as well as the risks of the individual domains, namely randomization process, deviations from intended interventions, missing outcome data, measurement of the outcome, and selection of the reported results. For cRCTs we also assessed bias arising from the identification or recruitment of participants into clusters (subdomain randomization) and additional questions such as awareness of being in a trial (participants and assessors), and outcome data availability for all clusters [36]. Based on the RoB 2 guidance [37] and its signaling questions, the overall risk and the risk of all subdomains was classified as *low risk*, *some concerns* or *high risk of bias*. Risk assessment was conducted by the review authors individually and discussed with the team to ensure robustness of the decisions (LS, JH, SV, FW, CJS).

### Synthesis method

Data for all included studies was synthesized narratively. For those studies reporting on anxiety, depressive symptoms and sleep disturbance, meta-analyses were conducted, and forest plots were created. Studies that reported effects of the interventions on resilience and mental or psychological wellbeing were summarized in a forest plot, but without pooling of the effect estimates due to heterogeneity in interventions and respective outcomes.

For the meta-analyses, we used unadjusted data as reported in the respective publications and applied the inverse variance method with a random-effects model. The inverse variance method was chosen to give smaller studies relatively more weight, and a random-effects model was deemed most appropriate in light of considerable heterogeneity. While we included one cRCT (no ICC reported) in the meta-analyses, sensitivity analyses were conducted with and without the respective study and with stepwise reductions in sample size of this study. Further, one three-armed RCT was included in the meta-analysis, for which we accounted by splitting the control group [38].

I^2^ was interpreted as an indicator of statistical heterogeneity among included studies. Further, we assessed heterogeneity by critical appraisal of study characteristics and descriptions of interventions, outcomes, and populations. Meta-analyses were conducted using RevMan 5.4.1. [39], which was also used to create the forest and funnel plots presented in this paper.

To categorize interventions and their components, we used the activity code framework by the Inter-Agency Standing Committee (IASC) Reference group on Mental Health and Psychosocial Support in Emergency Settings [40], which describes a four-tiered approach to address mental health needs during emergencies. The four tiers are the following: Basic services and security (for all), community and family support (for many/most), focused, non-specialized support (for some) and specialized services (for few). Specific activities and interventions are further categorized into eleven activity codes, namely information dissemination, facilitating community mobilization, community and family support, safe spaces, psychosocial support in education, supporting the inclusion of psychosocial considerations in other sectors and services, (case-focused) psychosocial work, clinical management of mental disorders by non-specialized health care providers, clinical management of mental disorders by specialized mental health care providers, and general activities [41].

Exploratory subgroup analyses were conducted according to (i) length and intensity of the intervention, (ii) integration of a physical activity component, (iii) IASC activity codes, (iv) presence/absence of personal interactions (between study participants and/ or with intervention providers) and (v) pre-selected study populations (elevated scores of anxiety or depressive symptoms). Given the small number of studies in these subgroups we report the respective effect sizes but decided against conducting statistical tests.

Sensitivity analyses were conducted to account for heterogeneity due to different comparators such as (i) no treatment or waitlist-control, (ii) active control, and (iii) alternative active control.

Data on the included RCT and cRCT protocols is presented in tabular form in Appendix D, Table A2.

### Effect measures

For the meta-analyses, we used standardized mean difference (SMD) for the effect size, as all included studies reported means and standard deviations. However, it has to be noted that different scales were used to measure the various outcomes such as anxiety and depressive symptoms. We report 95% confidence intervals and p-values with a significance level set at 0.05. Any non-null effects were of interest, and effects are reported and interpreted accordingly.

Effect estimates from studies that we excluded from the meta-analyses are presented as stated in the original publications.

### Reporting bias assessment

Reporting bias was assessed by critical appraisal of funnel plots for the outcomes anxiety and depressive symptoms. Funnel plots were created in RevMan [39] and assessed visually by additionally taking into account the small number and heterogeneity of included studies.

## Results

**Fig. 1:**
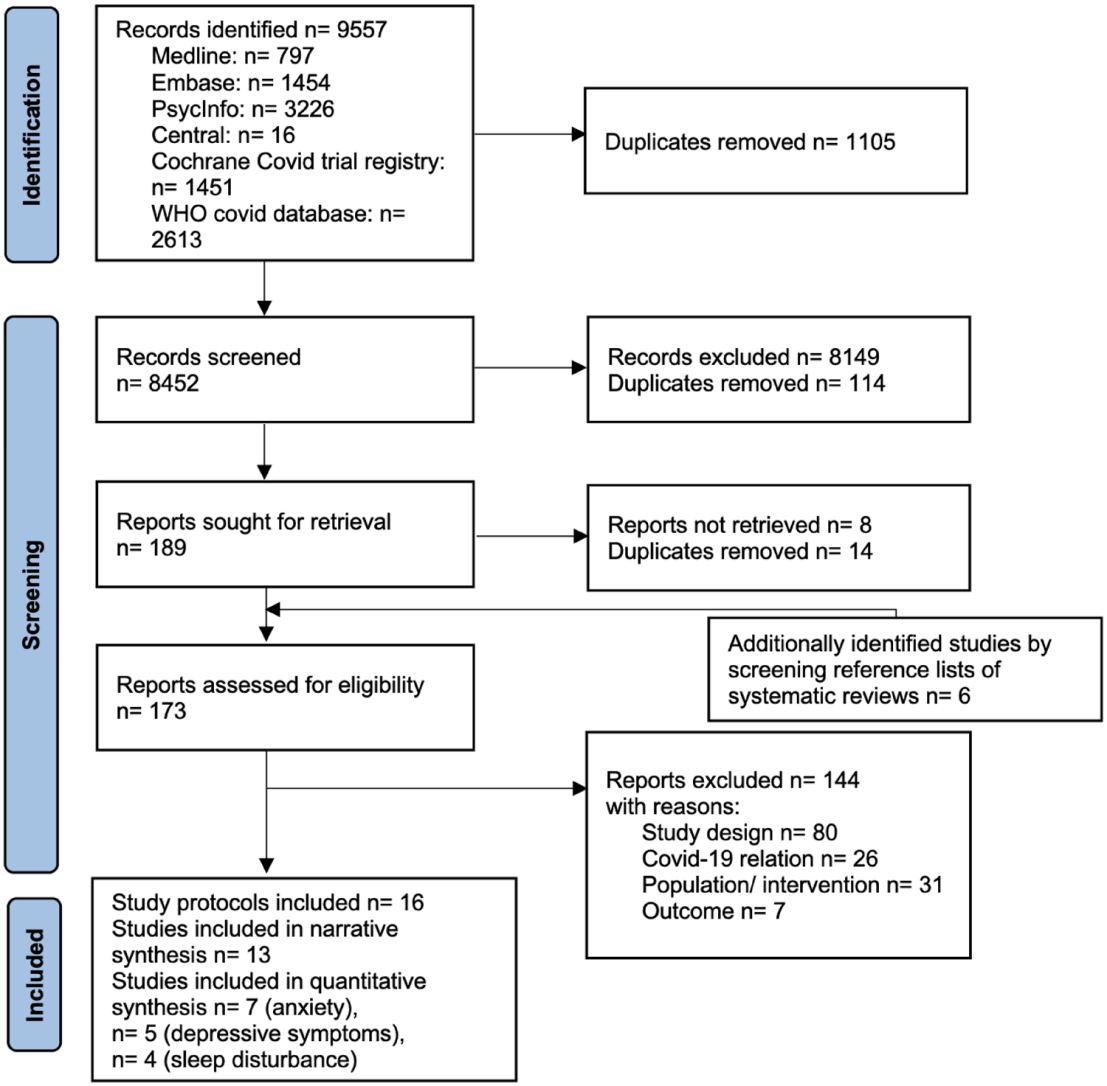
PRISMA Study flow

Our literature searches yielded 8452 unique records. After screening titles and abstracts, we assessed the full texts of the remaining 175 publications for eligibility. While we identified 16 study protocols (characteristics in Appendix D, Table A2), 13 studies were finally included in the narrative synthesis. Of these studies seven studies (eight interventions) reporting on anxiety [42–48] and five studies (six interventions) reporting on depression [43, 45–47, 49] were included in meta-analyses. Having more interventions than studies results from the inclusion of a three-arm RCT [46] whose distinct intervention arms were analyzed separately against a split control group. The PRISMA study flow is depicted in Fig. 1.

### Characteristics of included studies

Study characteristics are summarized in Table 1, funding sources of studies are listed in Appendix E, Table A3. Included studies were published between September 2020 and June 2022. Studies were conducted in Canada (n = 2) [45, 50], China (n = 6) [42, 43, 47, 48, 51, 52], Turkey (n = 2) [44, 49], the United Kingdom (n = 1) [53] and the United States (n = 2) [46, 54]. The participants comprised (elementary) school children [44, 50] and adolescents (> = 10 years) [42, 43, 45–49, 51–53], respectively. Although inclusion criteria were concerned with the general population, in five studies adolescents were selected based on elevated scores of anxiety, depressive symptoms or vulnerability [42, 43, 47, 51, 52] representing a higher risk population but without a clinical diagnosis.

**Table 1:** Characteristics of included studies

First author	Year	References	Country	Study design	n	Population age* mean (SD), range in years	Main intervention	Overall risk of bias
Altuntas	2022	[49]	Turkey	RCT	38	12.95 (0.65)	Callisthenic exercise	High
Chen	2021	[42]	China	RCT	72	14.4 (1.0) 13–16	Mindfulness meditation and aerobic exercises	High
Ding	2020	[43]	China	RCT	141	15.2 (2.1) 15.3 (2.4) 12–18	Health education, empowering for peer support, physical exercises	High
Karadag	2021	[44]	Turkey	RCT	178	9.07 (0.8)	Eye Movement Desensitization and Reprocessing (EMDR) derived self-help	High
Malboeuf- Hurtubise (art)	2021	[45]	Canada	cRCT	22	11.3 (4th and 5th grade)	Emotion-based directed drawing	High
Malboeuf- Hurtubise (philosophy)	2021	[50]	Canada	cRCT	37	8.18 (1st–5th grade)	Philosophy training	High
Pavarini	2021	[53]	UK	RCT	100	16.39 16–18	Peer support training	High
Schleider	2022	[46]	US	RCT (3 arms)	2452	13–16	Single session interventions (a) Behavioral activation (b) Mindset growth	High
Shao	2021	[51]	China	RCT	62	15.67 (1.01) 15.98 (0.11)	Group psychological intervention, dancing	High
Tymofiyeva	2022	[54]	US	RCT	21	16.0 (1.0) 15.7 (1.4) 14–18	Training for awareness, resilience and action (informed by mindfulness, yoga, psychotherapeutic approaches)	High
Xu	2021	[52]	China	RCT	84	12–19	Acceptance and commitment therapy and aerobic exercise	High
Zhang	2021	[47]	China	RCT	153	15.7 (2.05) 15.9 (1.07) 12–18	Psychological counseling and outdoor exercise	High
Zheng	2021	[48]	China	RCT	954	13.5 (0.5) 12–13	Peer-to-peer livestreaming app, health education, workout videos	Some concerns

*Reported separately for intervention and control group in some publications

*RCT* randomized-controlled trial, *cRCT* cluster-randomized-controlled trial

In summary, interventions were predominantly delivered online except in three studies [47, 51, 54]. These three studies implemented in-person interventions or components. Most interventions comprised group sessions [42, 43, 45, 47–54] while two interventions were self-guided [46] or parent-guided [44]. Apart from components that are based on different psychological approaches, six interventions included components promoting physical activity [42, 47–49, 51, 52] while one intervention involved an art intervention [45].

In regard to settings, six interventions were considered school-based [4, 42, 43, 45, 47, 48, 50] where interventions took place either at school or during (online) classes, or alternatively, participants were assigned to interventions based on their class affiliation (clustered study design). In regard to the IASC activity code classification, most of the interventions applied components belonging to codes 3 (strengthening community support, structured activities), code 5 (psychosocial support in education), and code 8 (psychological intervention).

Intensity and frequency of interventions varied widely between studies: in conclusion, four interventions took place once per week [45, 46, 50, 54] or several times per week (n = 9) [42–44, 47–49, 51–53]. The duration varied between one and five weeks in six studies [44–46, 48, 50, 53] and between seven and twelve weeks in seven studies [42, 43, 47, 49, 51, 52, 54]. More details on the interventions and the control conditions are presented in Table 2.

**Table 2:** Overview of interventions of included studies

First author	Intervention (short)	Description of intervention	IASC code	Duration/frequency	Control interventions	Mode of delivery (IG)	Outcomes*	Tools and results (IG;CG) (Mean, SD)	Follow up
Altuntas	Callisthenic exercise	Full body exercises, with warm-up and cool-down, provided by physiotherapist	3	45 min 3/week 8 weeks	No intervention	Online group	Anxiety, depression, sleep quality, quality of life	Beck Anxiety Scale: not reported in mean, SD Reynolds Adolescent Depression Scale: 50.26 (9.18); 53.63 (12.29) Pittsburgh Sleep Quality Index (PSQI): 2.84 (1.5); 4.16 (2.41) Pediatric Quality of Life Inventory (PedsQL) psychosocial: 1251.31 (196.7); 1207.9 (119.6)	8 weeks
Chen	Mindfulness meditation and aerobic exercises	Lectures and exercises for mindfulness meditation. Group meditation followed by exchange of experiences and feelings. Aerobic exercises (medium intensity) under guidance of coach	3,5,8	30 min 5/week 5 weeks	Health education on pandemic	Online group	Anxiety, positive and negative affect, wellbeing	Self-rating Anxiety Scale (SAS): 50.8 (9.3); 57.1 (8.9) Positive Affect Scale (PANAS): 32.7 (6.1); 29.2 (8.1) Negative Affect Scale (PANAS): 19.5 (4.2); 22.3 (6.3) Psychological Well-Being Scale (PWBS): 9.5 (1.9); 8.3 (2.7)	10 weeks
Ding	Health education, empowering for peer support, physical exercises	Health knowledge and behaviors through group discussion, role-playing, games. Task to educate their peers by organizing weekly peer-education seminars. Aerobic exercise of moderate intensity with sports watch	3,5	10 min 2/day 3/week 8 weeks	Health education on lifestyle, mental health and pandemic	Online group + self-guided	Anxiety, depression, sleep quality	SAS: 55 (10.49); 60.37 (9.65) Self-rating Depression Scale (SDS): 53.83 (9.52); 58.51 (9.63) PSQI: 5.63 (2.65); 7.37 (3.14)	8 weeks
Karadag	EMDR derived self-help	Booklet containing EMDR-derived techniques to be guided by parents	1,8	20 min 3/week 1 week	Waitlist	Digital material, parent guided	Anxiety, specific stress symptoms	State-Trait Anxiety Inventory for Children (STAIC): 28.5 (8.3); 29 (9.9) Childhood Posttraumatic Stress Reaction Index (CPTS-RI): not reported in mean, SD	4 weeks
Malboeuf- Hurtubise (art)	Emotion-based directed drawing	Drawing activities targeted towards exploring emotions (fear, worry, irritation) and feelings, followed by opportunity to share thoughts, feelings, reactions and discuss COVID-19. Guided by psychology students	3,5,8	45 min 1/week 5 weeks	Mandala drawing	Online group	Anxiety, depression, hyperactivity, inattention, mindfulness	Selected items from the Behavior Assessment Scale for Children-3rd edition (BASC III): anxiety: 3.5 (1.7); 2.87 (0.83) depression: 2.07 (1.49); 2.62 (1.5) hyperactivity: 1.78 (1.57); 2.0 (1.3) inattention: 1.23 (1.23); 2.12 (1.24) mindfulness: 2.04 (0.85); 2.3 (0.77)	6 weeks
Malboeuf- Hurtubise (philosophy)	Philosophy training	Topics covered: school and education, sadness and fear, personal freedom and rules, being old, death. Short video clips were used as primers. Led by psychology students	5, 7/8	60 min 1/week 1 week	Mindfulness-based intervention	Online group	Basic psychological needs satisfaction, mental health difficulties	Self-constructed items: 9.38 (2.18); 11.64 (2.57) Selected items from BASC III (anxiety/inattention): 3.87 (2.04); 5.0 (2.41)	6 weeks
Pavarini	Peer support training	Modules on establishing rapport, active listening, grief and trauma, confidentiality, self-care, coping strategies, crisis management, signposting and referrals and making a difference to the community	2,3	4 h 5/week 1 week	Waitlist	Online group	Emotional symptoms, mental wellbeing	Warwick–Edinburgh Mental Wellbeing Scale (WEMWBS): 55.56 (6.30); 45.04 (9.28) Strengths and Difficulties Questionnaire-Emotional symptoms subscale (SDQ-E): 2.84 (2.50); 4.36 (3.06)	1 week
Schleider	Single session interventions (SSI) Behavioural activation (BA) Growth mindset (GM)	BA: psychoeducation on depression, life values assessment, creation of activity action plan, writing exercise GM: introduction to the brain, testimonials and stories from older youths, information on personality change, writing exercise	8	20–30 min (once)	Supportive Therapy SSI: encourage participants to express emotions, no specific skills	Self-guided	Anxiety, Covid-19 related trauma symptoms, depression, hopelessness, perceived agency	Generalized Anxiety Disorder 7 (GAD-7): GM:2.68 (0.81); BA:2.73 (0.82); CG: 2.78 (0.79) Child Trauma Screen—Reaction Scale (CTS-RS): GM. 2.76 (0.66); BA: 2.73 (0.64); CG:2.83 (0.63) Children’s Depression Inventory (CDI): GM:11.58 (5.08); BA:11.47 (5.04); CG: 12.57 (4.97) Beck Hopelessness Scale-4 (BHS-4): GM:1.39 (0.83); BA:1.37 (0.8); CG: 1.51 (0.82) State Hope Scale (SHS): GM: 5.17 (1.65); BA: 5.06 (1.63); CG: 4.89 (1.64)	3 months
Shao	Group psychological intervention, dancing	Eight themes to learn about relationships to others and their own needs. Dance therapy included warm-up, using body language and present thoughts, assistance to express subjective experience, relaxing movement, by professional dance teacher	3,8	60 min 1/week 7 weeks	No intervention	In-person group	Anxiety and depression, life satisfaction, resilience	Anxiety and Depression Subscale of Achenbach Youth Self-Report: 1.55 (0.33); 2.99 (0.20) Healthy Kids Resilience Assessment: 4.02 (0.15); 2.48 (0.27) Satisfaction Scale: 5.46 (0.45); 2.93 (0.38)	8 weeks
Tymofiyeva	Training for awareness, resilience and action (informed by mindfulness, yoga, psychotherapeutic approaches)	Semi-manualized training informed by mindfulness, yoga-based techniques and modern psychotherapeutic approaches to target neurocircuitry involved in emotional hyperreactivity, agitation, dysphoric mood	8	1/week 12 weeks	No information	In-person and online group	Emotional wellbeing	SDQ-E: not reported in mean, SD	12 weeks^#^
Xu	Acceptance and commitment therapy (ACT) and aerobic exercise	Understanding about sudden public health emergencies and emotions, learning to make peace with emotions, accepting themselves incl. merits and demerits, using cognitive defusion, establish correct and active values, fighting for dreams, fulfilling commitments, provided by trained psychology students. Aerobic exercise: jogging, yoga, basketball and others	8	Exercise: 40–60 min 3/week 8 weeks, ACT: 40–50 min 1/week 6 weeks	Routine mental health education	Online group	Acceptance and action, wellbeing	Acceptance and Action Questionnaire 2nd Ed (AAQ-II) WEMWBS reported results contradictory, therefore not shown	8 weeks^#^
Zhang	Psychological counseling and outdoor exercise	Psychological training targeted at improving resilience. Including self-evaluation, exploring positive emotions, perceiving gratitude, cultivating positive and rational cognitive ways, strengthening psychological resilience, problem solving, helping, mutual trust etc Outdoor exercise: badminton, basketball, cross-country race, aerobics, etc	3,5,8	Counseling: 60 min 1/week 8 weeks Exercise: 50 min 2/week 8 weeks	Education on pandemic	In-person group	Anxiety, depression, resilience, sleep quality	SAS: 56.83 (10.96); 60.81 (9.51) SDS: 54.91 (9); 60.01 (8.87) Healthy Kids Resilience Assessment: 100.05 (7.88); 91.09 (9.57) PSQI: 6.21 (3.86); 7.64 (4.01)	8 weeks^#^
Zheng	Peer-to-peer live streaming app, SMS prompts, health education, workout videos	Capture short videos and photographs related to physical exercise or eye relaxation activities and share via live streaming or posting	3,5	4 × 15 min/ day 2 weeks	Health education, SMS prompts, workout videos	Online group	Anxiety, sleep quality	Spence Children's Anxiety Scale (SCAS): 3.49 (0.34); 3.79 (0.39) Pediatric sleep disturbance questionnaire: 2.57 (0.11); 2.55 (0.11)	2 weeks

*Only outcomes of relevance for this review listed, SD

#assumed follow-up

*IG* intervention group, *CG* control group, *SD* standard deviation

Control conditions comprised no treatment or waitlist-control [44, 49, 51, 53, 54], active control (predominantly health education) [4, 42, 43, 47, 48, 52] and alternative active control (e.g. Mandala drawing, mindfulness-based meditation, expressing emotions) [45, 46, 50].

### Risk of bias assessment

Risk of bias was considered “high” across all studies, except for one study with “some concerns” [48]. The overall high risk of bias was mainly due to missing information on the relevant items and domains such as randomization, participant selection, blinding, deviations from intended interventions, and selective reporting. In addition, one study presented contradictory results and tables [52] so that these results could not be included in quantitative synthesis. The risk of bias assessments for all included studies and RoB domains are shown in Appendix F, Figs. A1 and A2.

The funnel plots for the outcomes of anxiety and depressive symptoms (Appendix G, Figs. A3 and A4) showed the recommended inverted cone shape only to some extent. Taking into account considerable heterogeneity among studies and the small number of studies overall, we did not interpret the funnel plots as indicating systematic publication bias in our exploratory appraisal.

### Effects of interventions

#### Anxiety

**Fig. 2:**
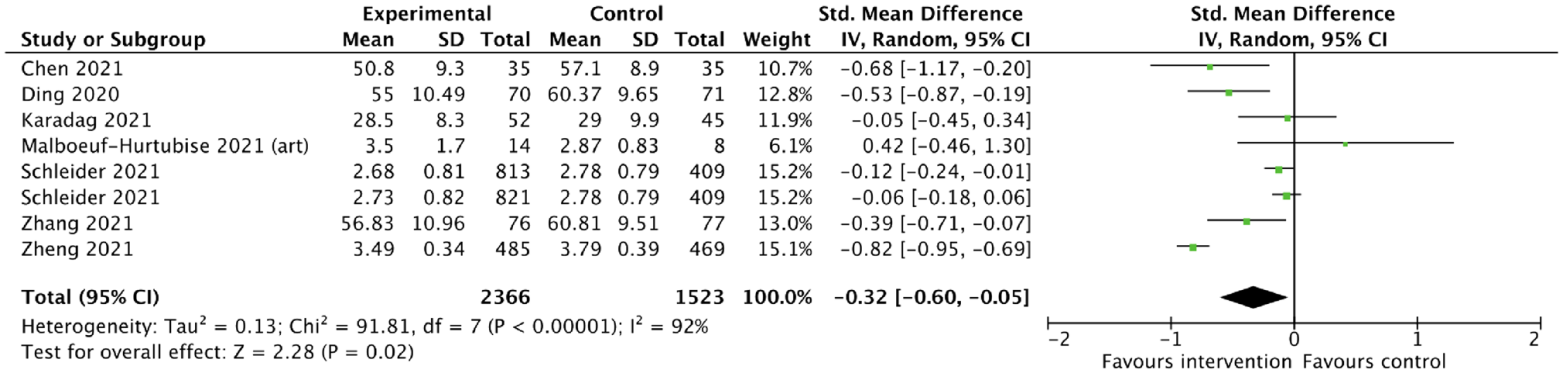
Combined effects (standardized mean difference) of various psychosocial interventions on anxiety among children and adolescents, measured using different anxiety scales (forest plot)

For studies reporting on anxiety that could be included in a meta-analysis (n = 7), we found evidence that children and adolescents in the intervention groups had significantly lower anxiety scores compared to the control groups. The pooled effect size of those studies that could be included in meta-analysis (n = 7) was SMD (95% CI) was − 0.32 (− 0.60, − 0.05), p < 0.01), the corresponding forest plot is presented in Fig. 2. Studies that could not be included in meta-analysis but reported on the same outcome also showed significant reductions in anxiety in the intervention groups compared to the control groups [49, 51].

#### Depressive symptoms

**Fig. 3:**
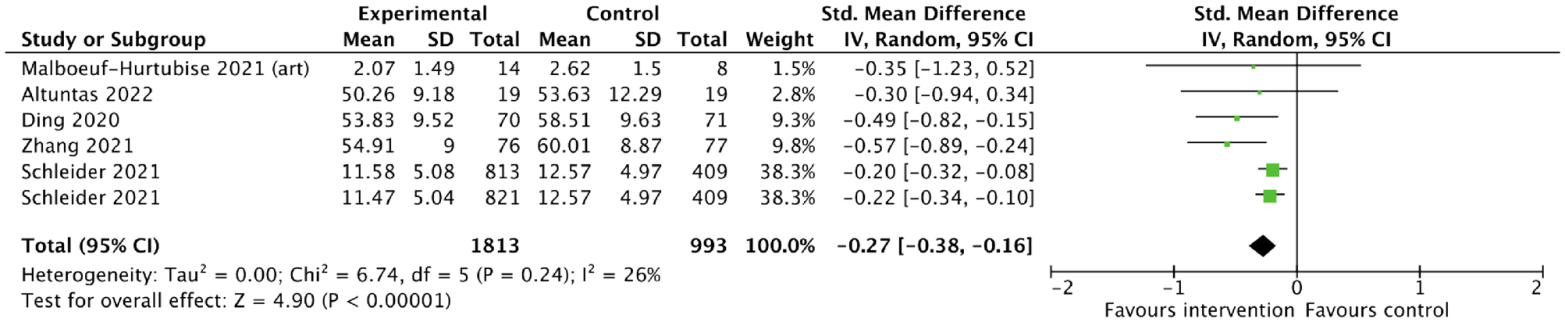
Combined effects (standardized mean difference) of various psychosocial interventions on depressive symptoms among children and adolescents, measured using different scales for depressive symptoms (forest plot)

Similarly, meta-analysis of studies reporting on depressive symptoms (n = 5) showed an overall positive intervention effect. The respective forest plot is depicted in Fig. 3. Overall, we found a reduced SMD (95% CI) of − 0.27 (− 0.38, − 0.16), p < 0.01) comparing the intervention and the control groups. The study that could not be included in meta-analysis but also reported on this outcome showed a significant reduction in depressive symptoms in the intervention group compared to the control group [51].

#### Sleep disturbance

While some studies found a decrease in sleep disturbance scores [43, 47, 49], one study reported higher sleep disturbance scores in the intervention group compared to the control group [48]. Therefore, pooling the effects of these four studies did not suggest a difference between intervention and control groups (SMD (95% CI): − 0.3 (− 0.79; 0.15), p = 0.19, I^2^ = 89%, forest plot see Appendix H, Figure A5).

#### Resilience and mental and psychological wellbeing

**Fig. 4:**
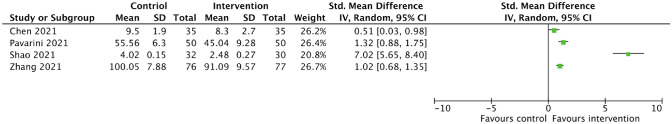
Effects of various psychosocial interventions (standardized mean difference) on resilience and wellbeing outcomes (forest plot, data of individual studies)

Narrative synthesis of studies analyzing resilience [42, 53], mental wellbeing [47] and psychological wellbeing [51] found a positive effect of interventions on the respective outcomes compared to controls (Fig. 4).

#### Further outcomes

Various other outcomes for children and adolescents were assessed in the included studies. Studies measuring life satisfaction [51], basic psychological needs satisfaction [50] and quality of life [49] reported improvements in the intervention group compared to the control group. Further, studies observed reductions in mental health difficulties [50], COVID-19-related trauma symptoms [46] (only growth mindset intervention), hopelessness [46] (only behavioral activation intervention), negative emotional symptoms [53], inattention [45] and posttraumatic stress [44] in the intervention groups compared to the control groups. No differences between groups were reported for hyperactivity and mindfulness [45] and psychosocial quality of life [49].

None of the included studies reported on outcomes of interest at the parent/caregiver-level.

### Sensitivity analyses

When excluding the only cRCT [45] from meta-analyses (outcome anxiety and outcome depressive symptoms) and when including the study with stepwise decreasing sample sizes, the pooled effect remained significant (data not shown). Moreover, the effect was larger compared to including the study without adjustments for the design effect.

Sensitivity analyses regarding different control conditions indicated a larger pooled effect for studies with an active control (e.g. health education) compared to studies with an alternative active control (e.g. expressing emotions, mandala drawing). Across the sensitivity analyses (data not shown) intervention effects remained significant.

Pooling of data from studies with a waitlist- or no treatment control was not feasible due to a limited number of studies with these control conditions (only one study on anxiety [44] and one on depressive symptoms [49]).

### Subgroup analyses

Interventions taking place more than once a week and over a longer period of seven to twelve weeks were associated with a greater reduction of anxiety and depressive symptoms (anxiety: SMD (95% CI): − 0.50 (− 0.71; − 0.29); depressive symptoms: SMD (95% CI): − 0.50 (− 0.72; − 0.28) compared to interventions which were implemented only once a week and over shorter periods of time (anxiety: SMD (95% CI): − 0.21 (− 0.56; − 0.14); depressive symptoms: SMD (95% CI): − 0.21(− 0.29; − 0.13)). More detailed results of the subgroup analyses and pooled effect sizes for the outcome depressive symptoms are presented in Appendix I, Table A4. Interventions with a physical activity component seemed to be more effective regarding a reduction in anxiety and depressive symptom scores than interventions without such a component: when only interventions with physical activity component were considered, the pooled SMD (95% CI) for anxiety was − 0.65 (− 0.95; − 0.35) compared to − 0.32 (− 0.60, − 0.05). For depressive symptoms effect sizes were – 0.51 (− 0.80; − 0.23) compared to − 0.27 (− 0.38; − 0.16) respectively. The results of the exploratory subgroup analyses further suggested a potentially different effectiveness of interventions according to their assigned activity codes. As such, school-based interventions (psychological support in education) and community and family support appeared to reduce anxiety and depressive symptoms more strongly (anxiety: SMD (95% CI): − 0.53 (− 0.82; − 0.25)) than psychological interventions (anxiety: SMD (95% CI): − 0.17 (− 0.32; − 0.02)). Across interventions that enable a personal interaction (between study participants and intervention providers and among study participants), we found a greater effect on anxiety and depressive symptoms compared to interventions that were self- or parent-guided. Studies directed specifically at children and adolescents with already elevated anxiety or depressive symptom scores showed a greater reduction of symptoms than studies targeting children and adolescents from an unselected general population.

## Discussion

This systematic review reports on interventions aimed at building resilience and/or ameliorating negative psychosocial effects of the COVID-19 pandemic on children and adolescents. Given the continuing knowledge gap on this topic three years after the pandemic, gathering evidence to identify effective and state of the art approaches to mitigate the harmful effects of crises on the young people is of paramount importance.

Within the scope of this review, we included 13 experimental studies reporting outcomes of anxiety, depressive symptoms, and sleep disturbance, as well as positive mental health outcomes (such as resilience).

Based on meta-analyses, we found evidence of reductions in anxiety and depressive symptoms in the intervention groups compared to control groups. The rather small effect sizes could possibly reflect the relatively short duration of follow-up.

Exploratory subgroup analyses of anxiety and depressive symptoms suggested higher effectiveness of interventions (i) delivered over a longer period of time and with a high frequency of sessions, (ii) allow for direct interaction with others, (iii) include a physical activity component, or (iv) are implemented via schools or in groups of classmates. Delivering interventions in school settings could be a favorable approach, as this allows easy access to children as a target group, while keeping children in their familiar peer-group [55, 56]. Shared experiences could enable better consolidation of learnt skills and behaviors and have the potential to enhance the effects of the intervention.

In general, most of the included studies comprised online group sessions where direct personal interaction was enabled to provide group experiences and social learning that were hindered by contact restrictions [1, 2]. In addition, our results suggest that interventions that include a physical activity component have a larger effect sizes than interventions that include only psychological counseling. However, it is important to consider the substantial overlap of studies that applied both intervention components fulfilling the criteria of a complex intervention. In addition, psychological counseling interventions might show more effectiveness in individuals with clinical diagnoses or highly elevated but subclinical symptom scores, which we have excluded from this review.

Although systematic reviews of mental health interventions for children and adolescents in other health emergencies or situations (indirect evidence) exist [17–19, 21–23, 25], direct evidence from studies conducted during the COVID-19 pandemic with its specific characteristics is accumulating slowly.

Overlapping concepts and components found in mental health interventions conducted before the pandemic included: physical activity [57, 58], yoga [20], art [59, 60], psychoeducation, psychological counseling, and psychotherapeutic approaches (e.g. CBT) [20], which were also found in the included studies in our review focusing on the COVID-19 pandemic.

Psychotherapeutic approaches, counseling and psychoeducation appear to be particularly promising approaches in times of the COVID-19 pandemic. Converting such interventions to online formats can be challenging [61–63]. However, compared to other interventions, they might be easier to implement.

Several research gaps and open questions still exist: while most studies used online platforms or video conferencing for interaction, only one study made use of an app [48]. This is noteworthy considering that many children worldwide own and regularly use smartphones. Additionally, transformative digital mental health concepts such as ecological momentary assessment (EMA) and ecological momentary interventions (EMI) are on the rise and based on apps/smartphones [64–66].

Only few studies allowed for major adaptations of the intervention to the need of the individual, as most interventions were group-based and followed a standardized program. Exceptions were i) counseling-based interventions that allowed individual topics to be addressed and ii) physical activity components that allowed the selection of specific games and exercises.

To address for specific contextual factors, an individualized stepped-care approach is increasingly advocated by experts. Depending on pre-existing characteristics or conditions, individuals receive treatment with varying intensity or content. In this review, five of the included studies explicitly focused on children or adolescents with elevated mental health symptoms. In one study [52] subgroups of children were considered according to different categories of subjective wellbeing and psychological distress trying to address individual needs and demands.

Referring to the IASC guideline [40] mental health interventions should focus on basic services for all, on community and family support for many, on focused, non-specialized support for some and for specialized services for few persons. With respect to the IASC activity codes [41], we found that most published interventions included components of categories 3 (family and community support, structured activities), 5 (psychosocial support in education, schools) and 8 (psychological intervention). Taking into account that we excluded individuals with pre-existing psychiatric diagnosis (in which case interventions would belong to code 9 and 10) and that the pandemic did not lead to disruptions to infrastructure such as housing, water supply, electricity (in which case interventions would most likely belong to code 6), it remains noteworthy that we hardly identified studies that included support for community emergency relief or communal spaces or meetings (activity code 2) or that focused on psychological first aid or linking vulnerable individuals to existing resources (activity code 7).

### Strengths and limitations

This systematic review followed a rigorous methodology based on best-practice standards for planning, conducting and reporting systematic reviews and meta-analyses. As part of the coverCHILD [67] research network, authors constisted of experts with diverse backgrounds and institutions in Germany from the clinical and public health sectors and related fields. The research question was consented by the interdisciplinary national consortium, which prioritizes important topics for steering health care and research focus in the future to ensure results that are relevant for interdisciplinary stakeholders. Another methodological strength of this review is the application of established frameworks, WHO guideline and models for development of the protocol and data extraction [27, 28, 31, 35, 40, 41]. Further, we were able to cover a multitude of languages for screening and data extraction given the large team of reviewers. Resilience as a recognized social construct can be understood in different ways. In the context of this systematic review, we followed the definition by Kalisch and colleagues. Both of the included studies that assessed resilience applied the standardized instrument “Healthy Kids Resilience Assessment” based on the work of Hu & Gan [68].

However, this review also comes with some limitations. One content related limitation is the exclusion of studies that focused exclusively on parental outcomes other than parental stress, parenting skills or resilience. Thus, this review was not designed to comment on or assess interventions targeting post-partum depression, transgenerational transmission of mental disorders or other specific contextual factors that could serve as potentially interesting targets of interventions.

Another limitation might be that we included only experimental study conditions (RCTs). Therefore, we might have missed evidence from other study designs. On the other hand, we could focus on the best possible quality of evidence to draw conclusions.

At the same time, incomplete reporting or non-adherence to guidelines for RCTs [69] was common, most likely attributable to circumstances related to the pandemic itself, such as short time frames for study planning and conceptualization, difficulties in recruitment and implementation due to contact restrictions or accelerated peer-review of scientific journals [70].

Limitations regarding the meta-analysis include considerable heterogeneity between interventions and the inclusion of studies with analyses conducted by intention-to-treat and per-protocol methods (which we decided to do given the generally high risk of bias of studies). Further, the small number of studies limited the assessment of publication bias by appraisal of funnel plots, as publication bias becomes more apparent with a larger number of studies. Apart from this, the small number of studies limits the informative value (validity) of comparisons of subgroup analyses. Therefore, results should be interpreted cautiously. Heterogeneity of interventions is a limitation to all our findings, not only limited to meta-analysis. Studies implemented different intervention components, theories, implementation schedules and directed at different populations (age, vulnerability, symptom groups). Therefore, drawing conclusions on which strategy (including which timing and sequence) is most suited or effective for which target group or which specific symptoms is not feasible based on our findings.

Regarding the categorization of interventions and their comparison, it is important to note that many interventions were complex interventions in nature that were assigned to several categories based on intervention delivery, content, and components, which impede predictions about the effectiveness of single components and categories.

### Implications for research

In conclusion, this project revealed still existing knowledge gaps about how to effectively address the psychosocial impact the pandemic posed and still poses on children and adolescents. While included studies indicated the effectiveness of certain interventions in reducing anxiety and depressive symptoms as well as to enhance resilience and other positive mental health outcomes, the informative value of future research would benefit from higher methodological quality and diligent reporting.

Interventions should be conducted and examined over a longer follow-up period to detect long-term effects and explore possibilities to increase effects through higher intensity/duration. In addition, different components of interventions should be examined for their combination, synergies and co-benefits (e.g. by dismantling designs).

Families with younger children (newborns, toddlers and infants) have faced particular difficulties and challenges from the COVID-19 pandemic. However, evidence is scarce, and given the major impact of early life experiences and the role of prevention among the young, more research should be conducted that targets this at-risk population.

### Implications for practice

Given the positive effects of identified interventions that facilitate (online) group sessions and that comprise a psychological and a physical activity component, such interventions could be made available and accessible to the broader population of children and adolescents during the pandemic and possibly during other stressful situations, crises and events. Interventions should be purposely designed to fit within and complement existing psychosocial support structures and provide linkages to existing psychosocial services.

While interventions that are self-guided (e.g. via apps) may be easier to implement and provide more flexibility to participants, interventions that facilitate personal interaction between peers and trainers may be more effective in mitigating the consequences that arise because of pandemic containment measures. Interventions delivered in school settings can enable reciprocal learning and consolidation of skills and behaviors in familiar settings and serve an overall mental health strategy on a larger scale that may not necessarily be crisis- or occasion-specific. Interventions that include structured social, creative, sportive or recreational activities in the school setting could be a promising approach to prevention and mitigation and could be implemented by teachers or other trained professionals.

Depending on the content and design of the intervention, children or adolescents who are vulnerable or already show sub-clinical mental health problems could benefit even more than individuals with a pre-existing high resilience. To address health disparities, special attention should be paid to this vulnerable group.

Recent developments in mental health interventions apply a transdiagnostic approach (focusing on psychopathological processes and symptoms across disorder categories) and rapid assessment of symptoms, in order to enable timely response and targeted interventions [64, 66]. Digital (e.g. smartphone-based) solutions can provide children and adolescents with momentary interventions that can be accessed from anywhere and at any time. Given the evolution of digital health and younger generation´s attachment to digital solutions, mental health interventions in the future should incorporate digital components and new technologies to be responsive to the expectations of children and adolescents. Although digital mental health interventions cannot replace personal interaction, they could be combined with a component of face-to-face or real-time interaction [66].

## Conclusions

To learn from the COVID-19 pandemic and prepare for future pandemics, existing concepts of mental health prevention should be adapted and new strategies should be developed and evaluated. While we seek to prepare for future crises, in the aftermath of COVID-19, it is important to maintain psychosocial support for children and adolescents as a particularly vulnerable group. Therefore, advocacy and research funding (i.e. for mental health surveillance) are crucial to prioritize child and adolescent mental health issues and implement effective and sustainable approaches that address their particular needs during crises.

## Supplementary Information

Below is the link to the electronic supplementary material.

Supplementary file1 (DOCX 314 KB)

## Data Availability

Data supporting reported results is included in the Appendix to some extent. Further data is available upon request.
