# Supplementary material for: Interventions to build resilience and to ameliorate negative psychosocial effects of the COVID-19 pandemic on children and adolescents: a systematic review and meta-analysis
Source: Eur Child Adolesc Psychiatry. 2023 Aug 13;33(11):3707–26. doi: 10.1007/s00787-023-02280-y (PMC11588790; doi:10.1007/s00787-023-02280-y)
Supplement: Supplementary file 1 — Supplementary file1 (DOCX 314 KB) [file 787_2023_2280_MOESM1_ESM.docx]

**Supplementary Materials:** The following supporting information is included: Appendix A: Search strategy; Appendix B, Table A1: Excluded studies (full-text screening) with reasons; Appendix C: Extracted data items; Appendix D, Table A2: Characteristics of included RCT and cRCT protocols; Appendix E, Table A3: Funding sources of included studies; Appendix F, Figure A1 and A2: Risk of bias summary and graph; Appendix G, Figure A3 and A4: Funnel plots; Appendix H, Figure A5: Combined effects (standardized mean difference) of various interventions on sleep disturbance (forest plot); Appendix I, Table A4: Subgroup analyses.

**Appendix A: Search strategy**

MEDLINE

1 exp coronavirus/ or exp coronavirus infections/ or covid*.mp. or ((corona* or corono*) adj1 (virus* or viral* or virinae*)).mp. or (coronavirus* or coronovirus* or coronavirinae*).mp. or (wuhan* or hubei* or huanan).mp. or (2019-ncov or 2019ncov or ncov2019 or ncov-2019).mp. or (covid-19 or covid19 or covid-19 or covid19).mp. or (hcov-19 or hcov19).mp. or (sars-cov-2 or sarscov-2 or sarscov2 or sars-cov2).mp. or (sars-cov-1 or sarscov-1 or sarscov1 or sars-cov1).mp. or (sarscov19 or sars-cov19 or sarscov-19 or sars-cov-19).mp. or 2019 novel*.mp. or (ncov or n-cov).mp. or cov.mp. or (((respiratory* adj2 (symptom* or disease* or illness* or condition*)) or seafood market* or food market*) adj10 (wuhan* or hubei* or china* or chinese* or huanan*)).mp. or ((outbreak* or wildlife* or pandemic* or epidemic*) adj1 (china* or chinese* or huanan*)).mp. or exp severe acute respiratory syndrome/ or severe acute respiratory syndrome.mp. or sars*.mp. or exp pandemics/ or pandem*.mp. or exp epidemics/ or epidemic*.mp. 446548

2 ((exp infant/ or infant*.mp. or (baby or babies).mp. or exp child/ or child*.mp. or toddler*.mp. or exp adolescent/ or adolescent*.mp. or teen*.mp. or minor*.mp. or youth.mp. or student*.mp. or pupil*.mp. or (paediatr* or pediatr*).mp. or exp parents/ or parent*.mp. or mother*.mp. or matern*.mp. or father*.mp. or patern*.mp. or exp family/ or famil*.mp. or exp grandparents/ or (grandparent* or grand-parent*).mp. or (grandmother* or grand-mother*).mp. or (grandfather* or grand-father*).mp. or exp parent-child relations/ or (parent* or mother* or father*).mp.) adj3 (child* or infant*).mp. adj3 (relation* or bond*).mp.) or exp maternal behavior/ or exp paternal behavior/ or ((parent* or matern* or patern*) adj3 behavio*).mp. or exp caregivers/ or (caregiv* or care-giv*).mp. or carer*.mp. or educator*.mp. [mp=title, abstract, original title, name of substance word, subject heading word, floating sub-heading word, keyword heading word, organism supplementary concept word, protocol supplementary concept word, rare disease supplementary concept word, unique identifier, synonyms] 268274

3 exp health education/ or (educat* adj3 (health or parent* or psych* or child* or service* or distan* or train* or program* or technolog* or physical* or continu* or support*)).mp. or (learn* adj3 (support* or supervis* or continu* or distan* or material* or program* or resource* or assist*)).mp. or exp health promotion/ or (promot* adj3 (mental health or skill*)).mp. or exp teaching/ or exp teaching materials/ or (teach* adj3 (material* or aid* or method* or program* or session* or assist* or activit*)).mp. or (assist* adj3 (child* or famil* or food* or nutrition*)).mp. or (train* adj3 (physical* or intellect* or support* or program* or resource* or exercis*)).mp. or exp exercise/ or (activ* adj3 (physical* or schedul*)).mp. or self-help groups/ or (support* adj3 (group* or psych* or social* or pedagogical* or network* or communit* or famil* or traditional or spiritual or religious)).mp. or (skill* adj3 (social* or emotional* or calm* or cop* or cognit* or digital*)).mp. or exp behavior therapy/ or (behavio* adj3 (train* or treat* or therap* or manag*)).mp. or ((psych* adj3 (interven* or service* or work or counsel* debrief*)) or ((build* or enhanc* or increase* or improv*) adj3 resilien*)).mp. or (therap* adj3 strateg*).mp. or psychoeducation*.mp. or (information adj3 dissemination).mp. or ((community adj3 (mobili* or organisation or organization or activity or action)) or (space adj3 (safe or child-friendly)) or psychotherapy or (structur* adj3 activit*) or basic counsel*).mp. 1821176

4 exp mental health/ or (mental adj3 health).mp. or ((psych* or mental* or emotion* or social* or child*) adj3 (well-being or wellbeing)).mp. or exp mental disorders/ or ((psych* or mental* or depress*) adj3 (disease* or disorder* or ill* or disturb* or symptom*)).mp. or exp stress, psychological/ or (stress* adj3 (psych* or mental* or emotion* or famil* or parent*)).mp. or exp anxiety disorders/ or ((stress* or anxiet*) adj3 disorder*).mp. or (anxiet* or anxious).mp. or exp depression/ or depress*.mp. or exp psychological trauma/ or ((psych* or mental* or emotion*) adj3 (trauma* or damag* or injur* or harm*)).mp. or exp stress disorders, post-traumatic/ or (posttraumatic or post-traumatic).mp. or PTSD*.mp. or exp psychological distress/ or ((psych* or emotion*) adj3 (distress* or tension)).mp. or exp sleep wake disorders/ or (sleep* adj3 (disorder* or disturb* or problem* or difficult* or pattern)).mp. or exp child abuse/ or (abus* adj3 (physical* or emotional* or substance* or drug* or alcohol* or sexual* or child*)).mp. or maltreat*.mp. or mistreat*.mp. or neglect*.mp. or exp domestic violence/ or ((domestic or famil* or partner or home) adj3 (viol* or abus* or conflict*)).mp. or exp self concept/ or (self adj1 (concept or aware* or confront* or efficacy or image or perception or rat* or control* or esteem)).mp. or exp child behavior/ or (behavio* adj3 (child* or infant* or disorder* or disturb* or problem*)).mp. or exp behavioral symptoms/ or exp quality of life/ or (quality adj3 life).mp. or QoL.mp. or ((suicid* adj3 (thought* or behavior* or ideat* or attempt*)).mp. or exp substance-related disorders/ or exp resilience, psychological/ or (psycholog* adj3 resilien*).mp.) or (eat* adj3 disorder).mp. or (parent* adj distress).mp. or (WEMWBS or SWEMWBS or Warwick-Edinburgh or WHO-5).mp.

5 1 and 2 and 3 and 4

6 limit 5 to last 3 years

Search conducted June 30, 2022 via Ovid®

Ovid MEDLINE(R) and Epub Ahead of Print, In-Process, In-Data-Review & Other Non-Indexed Citations and Daily <1946 to June 29, 2022>

EMBASE:

1 exp coronavirinae/ or exp coronavirus infection/ or covid*.mp. or ((corona* or corono*) adj1 (virus* or viral* or virinae*)).mp. or (coronavirus* or coronovirus* or coronavirinae*).mp. or (wuhan* or hubei* or huanan).mp. or (2019-ncov or 2019ncov or ncov2019 or ncov-2019).mp. or (covid-19 or covid19 or corvid-19 or corvid19).mp. or (hcov-19 or hcov19).mp. or (sars-cov-2 or sarscov-2 or sarscov2 or sars-cov2).mp. or (sars-cov-1 or sarscov-1 or sarscov1 or sars-cov1).mp. or (sarscov19 or sars-cov19 or sarscov-19 or sars-cov-19).mp. or 2019 novel*.mp. or (ncov or n-cov).mp. or cov.mp. or (((respiratory* adj2 (symptom* or disease* or illness* or condition*)) or seafood market* or food market*) adj10 (wuhan* or hubei* or china* or chinese* or huanan*)).mp. or ((outbreak* or wildlife* or pandemic* or epidemic*) adj1 (china* or chinese* or huanan*)).mp. or exp severe acute respiratory syndrome/ or severe acute respiratory syndrome.mp. or sars*.mp. or exp pandemic/ or pandem*.mp. or exp epidemic/ or epidemic*.mp. 551160

2 ((exp infant/ or infant*.mp. or (baby or babies).mp. or exp child/ or child*.mp. or toddler*.mp. or exp adolescent/ or adolescent*.mp. or teen*.mp. or minor*.mp. or youth.mp. or student*.mp. or pupil*.mp. or (paediatr* or pediatr*).mp. or exp parent/ or parent*.mp. or mother*.mp. or matern*.mp. or father*.mp. or patern*.mp. or exp family/ or famil*.mp. or exp grandparent/ or (grandparent* or grand-parent*).mp. or (grandmother* or grand-mother*).mp. or (grandfather* or grand-father*).mp. or exp child parent relation/ or (parent* or mother* or father*).mp.) adj3 (child* or infant*).mp. adj3 (relation* or bond*).mp.) or exp parental behavior/ or ((parent* or matern* or patern*) adj3 behavio*).mp. or exp caregiver/ or (caregiv* or care-giv*).mp. or carer*.mp. or educator*.mp. [mp=title, abstract, heading word, drug trade name, original title, device manufacturer, drug manufacturer, device trade name, keyword heading word, floating subheading word, candidate term word] 348129

3 exp health education/ or exp parenting education/ or exp psychoeducation/ or (educat* adj3 (health or parent* or psych* or child* or service* or distan* or train* or program* or technolog* or physical* or continu* or support*)).mp. or (learn* adj3 (support* or supervis* or continu* or distan* or material* or program* or resource* or assist*)).mp. or exp health promotion/ or (promot* adj3 (mental health or skill*)).mp. or exp teaching/ or (teach* adj3 (material* or aid* or method* or program* or session* or assist* or activit*)).mp. or (assist* adj3 (child* or famil* or food* or nutrition*)).mp. or exp training/ or (train* adj3 (physical* or intellect* or support* or program* or resource* or exercis*)).mp. or (activ* adj3 (physical* or schedul*)).mp. or exp support group/ or (support* adj3 (group* or psych* or social* or pedagogical* or network* or communit* or famil* or traditional or spiritual or religious)).mp. or (skill* adj3 (social* or emotional* or calm* or cop* or cognit* or digital*)).mp. or exp behaviour therapy/ or (behavio* adj3 (train* or treat* or therap* or manag*)).mp. or (psych* adj3 (interven* or service* or work or counsel* debrief*)).mp. or ((build* or enhanc* or increase* or improv*) adj3 resilien*).mp. or (therap* adj3 strateg*).mp. or psychoeducation*.mp. or (information adj3 dissemination).mp. or ((community adj3 (mobili* or organisation or organization or activity or action)) or (space adj3 (safe or child-friendly)) or psychotherapy or (structur* adj3 activit*) or basic counsel*).mp. 1887913

4 exp mental health/ or (mental adj3 health).mp. or exp psychological well-being/ or ((psych* or mental* or emotion* or social* or child*) adj3 (well-being or wellbeing)).mp. or exp mental disease/ or ((psych* or mental* or depress*) adj3 (disease* or disorder* or ill* or disturb* or symptom*)).mp. or exp mental stress/ or (stress* adj3 (psych* or mental* or emotion* or famil* or parent*)).mp. or exp anxiety disorder/ or ((stress* or anxiet*) adj3 disorder*).mp. or (anxiet* or anxious).mp. or exp depression/ or depress*.mp. or exp psychotrauma/ or ((psych* or mental* or emotion*) adj3 (trauma* or damag* or injur* or harm*)).mp. or exp posttraumatic stress disorder/ or (posttraumatic or post-traumatic).mp. or PTSD*.mp. or exp distress syndrome/ or ((psych* or emotion*) adj3 (distress* or tension)).mp. or exp sleep disorder/ or (sleep* adj3 (disorder* or disturb* or problem* or difficult* or pattern)).mp. or exp child abuse/ or (abus* adj3 (physical* or emotional* or substance* or drug* or alcohol* or sexual* or child*)).mp. or maltreat*.mp. or mistreat*.mp. or neglect*.mp. or exp domestic violence/ or ((domestic or famil* or partner or home) adj3 (viol* or abus* or conflict*)).mp. or exp self concept/ or (self adj1 (concept or aware* or confront* or efficacy or image or perception or rat* or control* or esteem)).mp. or exp behavior disorder/ or (behavio* adj3 (disorder* or disturb* or problem*)).mp. or exp child behavior/ or (behavio* adj3 (child* or infant*)).mp. or exp substance abuse/ or (quality adj3 life).mp. or qol.mp. or ((suicid* adj3 (thougth* or ideat* or behavior* or attempt*)).mp. or exp psychological resilience/ or (psychologic* adj3 resilien*).mp. or (eat* adj3 disorder).mp. or (parent* adj distress).mp. or (WEMWBS or SWEMWBS or Warwick-Edinburgh or WHO-5).mp.) 3936161

5 1 and 2 and 3 and 4 1705

6 limit 5 to last 3 years 1454

Search conducted June 30, 2022 via Ovid®

Embase <1988 to 2022 Week 25>

PsycInfo

( TX coronavirus OR TX “coronavirus infection” OR TX covid* OR TX ((corona* or corono*) n1 OR (virus* or viral* or virinae*)) OR TX (coronavirus* or coronovirus* or coronavirinae*) OR TX (wuhan* or hubei* or huanan) OR TX (2019-ncov or 2019ncov or ncov2019 or ncov-2019) OR TX (covid-19 or covid19 or corvid-19 or corvid19) OR TX (hcov-19 or hcov19) OR TX (sars-cov-2 or sarscov-2 or sarscov2 or sars-cov2) OR TX (sars-cov-1 or sarscov-1 or sarscov1 or sars-cov1) OR TX (sarscov19 or sars-cov19 or sarscov-19 or sars-cov-19) OR TX “2019 novel*” OR TX (ncov or n-cov) OR TX cov OR TX (((respiratory* n2 (symptom* or disease* or illness* or condition*)) or seafood market* or food market*) n10 (wuhan* or hubei* or china* or chinese* or huanan*)) OR TX ((outbreak* or wildlife* or pandemic* or epidemic*) n1 (china* or chinese* or huanan*)) OR TX sars* OR TX pandem* OR TX epidemic* ) AND ( TX infant* OR TX (baby or babies) OR TX child* OR TX toddler* OR TX adolescent* OR TX teen* OR TX minor* OR TX youth OR TX student* OR TX pupil* OR TX (paediatr* or pediatr*) OR TX parent* OR TX mother* OR TX matern* OR TX father* OR TX patern* OR TX famil* OR TX (grandparent* or grand-parent*) OR TX (grandmother* or grand-mother*) OR TX (grandfather* or grand-father*) OR TX ((parent* or mother* or father*) n3 (child* or infant*) n3 (relation* or bond*)) OR TX ((parent* or matern* or patern*) n3 behavio*)) OR TX (caregiv* or care-giv*) OR TX carer* OR TX educator* ) AND ( TX (educat* n2 (health or parent* or psych* or child* or service* or distan* or train* or program* or technolog* or physical* or continu* or support*)) OR TX (learn* n3 (support* or supervis* or continu* or distan* or material* or program* or resource* or assist*)) OR TX “health promotion” OR TX (promot* n3 (mental health or skill*)) OR TX teaching OR TX (teach* n3 (material* or aid* or method* or program* or session* or assist* or activit*)) OR TX (assist* n3 (child* or famil* or food* or nutrition*)) OR TX training OR TX (train* n3 (physical* or intellect* or support* or program* or resource* or exercis*)) OR TX (activ* n3 (physical* or schedul*)) OR TX (support* n3 (group* or psych* or social* or pedagogical* or network* or communit* or famil* or traditional or spiritual or religious)) OR TX (skill* n3 (social* or emotional* or calm* or cop* or cognit* or digital*)) OR TX (behavio* n3 (train* or treat* or therap* or manag*)) OR TX (psych* n3 (interven* or service*)) OR TX ((build* or enhanc* or increase* or improv*) adj3 resilien*) OR TX (information n3 dissemination) OR TX (community n3 (mobili* or organisation or organization or activity or action) OR TX (space n3 (safe or child-friendly)) OR TX (psychotherapy) OR TX (structur* n3 activit*) OR TX (basic counsel*) ) AND ( TX (mental n3 health) OR TX ((psych* or mental* or emotion* or social* or child*) n3 (well-being or wellbeing)) OR TX ((psych* or mental* or depress*) n3 (disease* or disorder* or ill* or disturb* or symptom*)) OR TX (stress* n3 (psych* or mental* or emotion* or famil* or parent*)) OR TX ((stress* or anxiet*) n3 disorder*) OR TX (anxiet* or anxious) OR TX depress* OR TX ((psych* or mental* or emotion*) n3 (trauma* or damag* or injur* or harm*)) OR TX (posttraumatic or post-traumatic) OR TX PTSD* OR TX ((psych* or emotion*) n3 (distress* or tension)) OR TX (sleep* n3 (disorder* or disturb* or problem* or difficult* or pattern)) OR TX (abus* n3 (physical* or emotional* or substance* or drug* or alcohol* or sexual* or child*)) OR TX maltreat* OR TX mistreat* OR TX neglect* OR TX ((domestic or famil* or partner or home) n3 (viol* or abus* or conflict*)) OR TX (self n1 (concept or aware* or confront* or efficacy or image or perception or rat* or control* or esteem)) OR TX (behavio* n3 (disorder* or disturb* or problem*)) OR TX (behavio* n3 (child* or infant*)) OR TX (qol) OR TX (quality n3 life) OR TX (suicid* n3 (thougth* or ideat* or behavior* or attempt*) OR TX (psychologic* n3 resilien*) OR TX (eat* n3 disorder) OR TX (parent* n3 distress) OR TX (WEMWBS or SWEMWBS or Warwick-Edinburgh or WHO-5) )

CENTRAL

Date Run: 30/06/2022 02:21:43

Comment:

ID Search Hits

#1 MeSH descriptor: [SARS-CoV-2] explode all trees 985

#2 MeSH descriptor: [COVID-19] explode all trees 1852

#3 MeSH descriptor: [Child] explode all trees 61335

#4 MeSH descriptor: [Adolescent] explode all trees 110231

#5 MeSH descriptor: [Infant] explode all trees 34864

#6 MeSH descriptor: [Parents] explode all trees 5932

#7 MeSH descriptor: [Caregivers] explode all trees 2573

#8 MeSH descriptor: [Mothers] explode all trees 2174

#9 MeSH descriptor: [Fathers] explode all trees 219

#10 MeSH descriptor: [Students] explode all trees 5274

#11 MeSH descriptor: [Grandparents] explode all trees 18

#12 MeSH descriptor: [Mental Health] explode all trees 1929

#13 MeSH descriptor: [Mental Disorders] explode all trees 81311

#14 MeSH descriptor: [Substance-Related Disorders] explode all trees 16322

#15 MeSH descriptor: [Emotional Abuse] explode all trees 0

#16 MeSH descriptor: [Psychological Distress] explode all trees 260

#17 MeSH descriptor: [Resilience, Psychological] explode all trees 302

#18 MeSH descriptor: [Child Abuse] explode all trees 580

#19 MeSH descriptor: [Sleep Wake Disorders] explode all trees 9191

#20 MeSH descriptor: [Quality of Life] explode all trees 28699

#21 MeSH descriptor: [Suicidal Ideation] explode all trees 653

#22 MeSH descriptor: [Suicide, Attempted] explode all trees 479

#23 MeSH descriptor: [Emotional Regulation] explode all trees 100

#24 MeSH descriptor: [Health Education] explode all trees 21304

#25 MeSH descriptor: [Behavioral Disciplines and Activities] explode all trees 60313

#26 MeSH descriptor: [Parent-Child Relations] explode all trees 2157

#27 MeSH descriptor: [Father-Child Relations] explode all trees 74

#28 MeSH descriptor: [Mother-Child Relations] explode all trees 876

#29 MeSH descriptor: [Psychosocial Support Systems] explode all trees 65

#30 MeSH descriptor: [Psychosocial Functioning] explode all trees 21

#31 MeSH descriptor: [Internet-Based Intervention] explode all trees 351

#32 #1 or #2 1857

#33 #3 or #4 or #5 or #6 or #7 or #8 or #9 or #10 or #11 165066

#34 #12 or #13 or #14 #or #15 or #16 or #17 or #18 or #19 or #20 or #21 or #22 or #23 or #30 110935

#35 #24 or #25 or #26 or #27 or #28 or #29 or #31 78065

#36 #32 and #33 and #34 and #35 16

Cochrane COVID-19 Study Register

parent* OR child* OR adolescent* OR caregiver* OR famil*

activit* OR resilien* OR psychologic* OR psychosocial* OR support* OR mental OR counsel*

WHO COVID-19 Global literature on coronavirus disease

(tw:(infant*) OR tw:((baby OR babies)) OR tw:(child*)or tw:(toddler*) OR tw:(adolescent*) OR tw:(teen*) OR tw:(minor*) OR tw:(youth) OR tw:(student*) OR tw:(pupil*) OR tw:((paediatr* OR pediatr*)) OR tw:(parent*) OR tw:(mother*) OR tw:(matern*) OR tw:(father*) OR tw:(patern*) OR tw:(famil*) OR tw:((grandparent* OR “grand-parent*”)) OR tw:((grandmother* OR “grand-mother*”)) OR tw:((grandfather* OR “grand-father*”)) OR tw:((caregiv* OR “care-giv*”)) OR tw:(carer*) OR tw:(educator*)) AND (tw:(educat*) OR tw:(learn*) OR tw:(“health promotion”) OR tw:(teach*) OR tw:(assist*) OR tw:(train*) OR tw((“physical activity” OR “activity schedule”)) OR tw:(support*) OR tw:(skill*) OR tw:(behavio*) OR tw:((“psychological intervention*” OR “psychological service*”)) OR tw:(resilien*) OR tw:(psychoeducation) OR tw:(psychosocial) OR tw:(counsel*) OR tw:(“safe space”) OR tw:(“structured activit*”)) AND (tw:(“mental health”) OR tw:((“psychological well*” OR “mental well*” OR “emotional well*” OR “social well*” OR “child wel*”)) OR tw:((“mental disease” OR “mental disorder” OR “mental illness”)) tw:(stress*) OR tw:((anxiet* OR anxious)) OR tw:(depress*) OR tw:(trauma*) OR tw:(ptsd*) OR tw:(“psychological distress*”) OR tw:(“sleep disorder”) OR tw:(abus*) OR tw:(maltreat*) OR tw:(mistreat*) OR tw:(neglect*) OR tw:((“domestic violence” OR “family violence” OR “family conflict” OR “partner violence”)) OR tw:((“self-concept” OR “self-awareness” OR “self-efficacy” OR “self-perception” OR “self-control*” OR “self-esteem”)) OR tw:(resilien*) OR tw:(“psychological resilience”) OR tw:(“quality of life”) OR tw:(suicid*) OR tw:(“eating disorder”) OR tw:(“parenting distress”) OR tw:(wemwbs OR swemwbs OR warwick-edinburgh OR who-5)) AND db:("EuropePMC" OR "PREPRINT-MEDRXIV" OR "ProQuest Central" OR "Scopus" OR "Web of Science" OR "Academic Search Complete" OR "COVIDWHO" OR "LILACS" OR "GREY-COVIDWHO" OR "ELSEVIER" OR "Centers for Disease Control and Prevention" OR "National Technical Information Service" OR "Homeland Security Digital Library" OR "International HTA Db" OR "Social Science Open Access Repository" OR "International HTA Database" OR "TRID Database" OR "WHOIRIS" OR "Transportation Research Board" OR "BDENF" OR "COLNAL" OR "BINACIS" OR "CUMED" OR "BBO" OR "SES-SP" OR "INDEXPSI" OR "UNISALUD" OR "colecionaSUS" OR "BNUY" OR "LIPECS" OR "LIVECS" OR "UY-BNMED" OR "BISSAL" OR "CONASS" OR "SESSP-IDPCPROD" OR "BIGG" OR "BRISA" OR "ICTRP") AND type_of_study:("rct" OR "observational_studies" OR "review" OR "clinical_trials" OR "systematic_reviews" OR "policy_brief") AND la:("en" OR "es" OR "zh" OR "ru" OR "de" OR "fr" OR "it")

**Appendix B: Excluded studies**

**Table A1.** Excluded studies (full-text screening) with reasons

| **First author** | **Year** | **Title** | **Journal** | **Reason for exclusion** |
| --- | --- | --- | --- | --- |
| Zengin | 2021 | The effect of online solution-focused support program on parents with high level of anxiety in the COVID-19 pandemic: A randomised controlled study | Int J Clin Pract. 75: e14839. | Outcome |
| Agazzi | 2022 | Pandemic parenting: A pilot study of in-person versus internet-DOCS K-5 for caregivers of school-age children with disruptive behaviors | Clinical Child Psychology and Psychiatry. 27(3): 569–585 | Study design |
| Agazzi | 2021 | A nonrandomized trial of a behavioral parent training intervention for parents with children with challenging behaviors: In-person versus internet-HOT DOCS | Clinical Child Psychology and Psychiatry. 26(4): 1076–1088 | Study design |
| Aleyne | 2021 | Finding purpose in the COVID-19 pandemic: Partnering of child psychiatrists and educators for children´s health and academic success | Journal of the American Academy of Child & Adolescent Psychiatry. 60(10S): S43 | Study design |
| Arnold | 2021 | Treating Children Exposed to Domestic Violence: Group-Based Intervention | International Journal of Group Psychotherapy  https://doi.org/10.1080/00207284.2020.1856668 | Study design |
| Arnold | 2020 | A brief transdiagnostic pandemic mental health maintenance intervention | Counselling psychology quarterly. 34(3-4): 331–351 | Study design |
| Arshad | 2021 | Psychological Support Application Using The Real-Time Webrtc: A Case Study Of Undergraduate In Malaysian And Indonesian Higher Learning Institutions | Turkish Journal of Computer and Mathematics Education. 12(5): 1692-1698 | Population/ Intervention |
| Askar | 2021 | Evaluating the efficacy of child anxiety tales with an at-risk population of school-aged children: an online parent-administered intervention | Dissertation (Michigan State University, School of Psychology) | COVID-19 |
| Australian Catholic University | 2021 | I’ll be OK In Year 7: effects of the 8 week transition programs on Year 6 students' psychological well-being and successful transition to Year 7, during the COVID-19 pandemic | ACTRN12621001254886 | COVID-19 |
| Azad | 2021 | Psychotherapy of Children in the Age of COVID-19 | Psychiatry. 84(2): 134-136 | Study design |
| Baeza-Hernandez | 2022 | Pilot implementation of psychoeducational workshops on behavior management and stress management for parents and teachers | Pre-print doi: 10.31234/osf.io/mwv3k | Study design |
| Ballester-Ferrer | 2022 | COVID-19 Quarantine Impact on Wellbeing and Cognitive Functioning During a 10-Week High-Intensity Functional Training Program in Young University Students | Frontiers in Behavioral Neuroscience. 16: 822199 | Population/ Intervention |
| Batchelor | 2020 | Debate: Lessons learned in lockdown – a one-day remotely delivered training on low-intensity psychological interventions for common mental health conditions | Child and Adolescent Mental Health. 25(3): 175-177 | Study design |
| Bates | 2021 | Virtual Sport‐Based Positive Youth Development During the COVID‐19 Pandemic | Child and Adolescent Social Work Journal. 38: 437–448 | Study design |
| Behrens | 2022 | Child internalizing symptoms during the COVID-19 pandemic among maltreating and non-maltreating families: Examining the effects of family resources and the Reminiscing and Emotion Training intervention | Child Abuse & Neglect. 130: 105375 | COVID-19 |
| Behzadnia | 2021 | A Self-Support Approach to Satisfy Basic Psychological Needs During Difficult Situations | Motiv Emot. https://doi.org/10.1007/s11031-022-09968-9 | Population/ Intervention |
| Chen | 2020 | An online solution focused brief therapy for adolescent anxiety during the novel coronavirus disease (COVID-19) pandemic: a structured summary of a study protocol for a randomised controlled trial | Trials. 21: 402 | Population/ Intervention |
| Braccio | 2021 | Innovative use of videoconference tool for parental education during COVID-19 | Arch Dis Child. 106(Suppl 1): A1–A514 | Study design |
| Brog | 2021 | An internet-based self-help intervention for people with psychological distress due to  COVID-19: study protocol for a randomized controlled trial | Trials. 22: 171 | Population/ Intervention |
| Bruett | 2022 | Development of evidence-informed bridge programming to support an increased need for eating disorder services during the COVID-19 pandemic | Journal of Eating Disorders. 10: 71 | Study design |
| Caldwell | 2022 | The Effect of an After-School Physical Activity Program on Children’s Cognitive, Social, and Emotional Health during the COVID-19 Pandemic in Nova Scotia | Int. J. Environ. Res. Public Health. 19: 2401 | Study design |
| Canavese | 2021 | Massive Open Online Courses as Strategies to Address Violence through the Training of Health and the Intersectoral Professionals in Brazil | Journal of Aggression, Maltreatment & Trauma. 31(6): 769-778 | Study design |
| Casu | 2021 | Promoting Mental Health and Well-Being among Adolescent Young Carers in Europe: A Randomized Controlled Trial Protocol | Int J Environ Res Public Health. 18: 2045. | COVID-19 |
| Carlson | 2020 | Stay Active Physical Activity Program | NCT04675658  https://clinicaltrials.gov/ct2/show/NCT04675658 | Outcome |
| Cipriano | 2022 | Non-Suicidal Self-Injury: A School-Based Peer Education Program for Adolescents During COVID-19 Pandemic | Frontiers in Psychiatry. 12: 737544 | Study design |
| Cohen | 2021 | The Online Adaptation and Outcomes of a Family-Based Intervention Addressing Substance Use Disorders | Research on Social Work Practice. 31(3): 244-253 | COVID-19 |
| De Candia | 2021 | The exercise dependence at the time of COVID-19 pandemic: The role of psychological stress among adolescents | Journal of Human Sport and Exercise. 16(4proc): S1937-S1945 | COVID-19 |
| Devassy | 2021 | REaCH-Resiliency Engagement and Care in Health; a Befriending Intervention to Address the Psycho-Social Challenges of Vulnerable Youth in the Context of COVID-19 Pandemic: An Exploratory Trial in India | Sustainability. 13: 12920 | Population/ Intervention |
| Dominguez-Rodriguez | 2021 | A Self-Applied Multi-Component Psychological Online Intervention Based on UX, for the Prevention of Complicated Grief Disorder in the Mexican Population During the COVID-19 Outbreak: Protocol of a Randomized Clinical Trial | Frontiers in Psychology. 12: 644782 | Population/ Intervention |
| Ebersole | 2021 | Using the Community of Inquiry Framework to Examine Instructor Strategies for Emergency Remote Online Teaching during the COVID-19 Pandemic | Education Dissertations. 62:  https://digitalcommons.spu.edu/soe_etd/62 | Study design |
| Edeh | 2022 | Supporting business educators and students against COVID-19 trauma using trauma-focused cognitive behavioral therapy | Medicine. 101:14 | Population/ Intervention |
| El-Khani | 2022 | Caring for Your Child during COVID-19—Utilizing a Light-Touch Parenting Resource during Lockdown in Indonesia | Int J Environ Res Public Health. 19: 4046. | Study design |
| Elmer | 2021 | Rural Community Health: Keeping Michigan's Upper Peninsula Informed During the COVID‐19 Pandemic | The FASEB Journal. 35(Suppl 1).  https://doi.org/10.1096/fasebj.2021.35.S1.04567 | Study design |
| Eveleigh | 2022 | How Did Educators of Students with Learning Differences Use Social–Emotional Learning to Support Their Students and Themselves Early in the COVID-19 Pandemic? | Children & Schools. 44(1): 27-38 | Population/ Intervention |
| Fabriz | 2021 | Impact of Synchronous and Asynchronous Settings of Online Teaching and Learning in Higher Education on Students’ Learning Experience During COVID-19 | Frontiers in Psychology. 12: 733554 | Study design |
| Farell | 2022 | A Stage 1 Pilot Cohort Exploring the Use of EMDR Therapy as a Videoconference Psychotherapy During COVID-19 With Frontline Mental Health Workers: A Proof of Concept Study Utilising a Virtual Blind 2 Therapist Protocol | Frontiers in Psychology. 13: 901855 | Study design |
| Feinberg | 2022 | Long‐term Effects of Adolescent Substance Use Prevention on Participants, Partners, and their Children: Resiliency and Outcomes 15 Years Later During the COVID‐19 Pandemic | Prevention Science. https://doi.org/10.1007/s11121-022-01384-2 | COVID-19 |
| Feinberg | 2021 | Building long-term family resilience through universal prevention: 10-year parent and child outcomes during the COVID-19 pandemic | Family Process. 61: 76-90 | COVID-19 |
| Felsen | 2021 | "Web-based, second-best togetherness": Psychosocial group intervention with children of Holocaust survivors during COVID-19 | Am J Orthopsychiatry. 91(2):1 71-180 | Study design |
| Figgins | 2021 | The Effects of a College Wellness Course on Student’s Stress, Anxiety and Physical Activity Levels During the COVID-19 Pandemic | Dissertation proposal, Concordia University, Chicago | Study design |
| Fix | 2022 | An Evaluation of Building Our Nation’s Daughters (BOND): Improving Black Single Mother–Daughter Relationships and Well-Being | Journal of Child and Family Studies. 31: 237–246 | Population/ Intervention |
| Fleming | 2021 | Introduction of video messaging to a tertiary neonatal nit during COVID-19: the staff and parent experience | Arch Dis Child. 106(Suppl 1): A1–A514 | Study design |
| Francica | 2020 | Did You Receive the Post? Heideggerian leaps into online therapy with children during the COVID-19 pandemic | Journal of The Society for Existential Analysis. 31(2): 237-246 | Study design |
| Garcia | 2021 | Rapid, Full‐Scale Change to Virtual PCIT During the COVID‐19 Pandemic: Implementation and Clinical Implications | Prevention Science. 22: 269–283 | Study design |
| Giallo | 2021 | Family Foundations To promote parent mental health and family functioning during the COVID-19 pandemic in Australia: A mixed methods evaluation | Journal of Family Studies. https://doi.org/10.1080/13229400.2021.2019606 | Study design |
| Godara | 2021 | Investigating differential effects of socio- emotional and mindfulness-based online interventions on mental health, resilience and social capacities during the COVID-19 pandemic: The study protocol | PLoS ONE. 16(11): e0256323. | Population/ Intervention |
| Fayazi | 2021 | Evaluation of the effectiveness of prenatal self-care training application in Covid 19 pandemic on quality of life, depression, corona anxiety and fear of childbirth in pregnant women | IRCT20210531051457N1  https://www.irct.ir/trial/56585 | Outcome |
| Gonsalves | 2021 | A Guided Internet-Based Problem-Solving Intervention Delivered Through Smartphones for Secondary School Pupils During the COVID-19 Pandemic in India: Protocol for a Pilot Randomized Controlled Trial | JMIR Res Protoc. 10(10): e30339. | COVID-19 |
| Gray | 2022 | Harnessing Virtual Mom Power: Process and Outcomes of a Pilot Telehealth Adaptation of a Multifamily, Attachment-Based Intervention | Journal of Infant, Child, and Adolescent Psychotherapy. 21(1): 6-18 | Study design |
| Green | 2022 | Evaluation of an Augmented Cognitive Behavioural Group Therapy for Perinatal Generalized Anxiety Disorder (GAD) during the COVID-19 Pandemic | J Clin Med. 11: 209 | Study design |
| Guillén | 2022 | Effectiveness of family connections intervention for family members of persons with personality disorders in two different formats: Online vs face-to-face | Internet Interventions. 28: 100532 | COVID-19 |
| Kakasci | 2022 | A creative and practical approach to postpartum discharge education: Pecha kucha training via smart phone | Health Care for Women International. https://doi.org/10.1080/07399332.2022.2043860 | COVID-19 |
| Guruge | 2021 | Description of a Telephone and Internet-based Intervention to Improve Community Responses to COVID-19 Spread | Research Square. https://doi.org/10.21203/rs.3.rs-526751/v1 | Study design |
| Guzick | 2022 | Brief, parent-led, transdiagnostic cognitive-behavioral teletherapy for  youth with emotional problems related to the COVID-19 pandemic | Journal of Affective Disorders. 301: 130–137 | Study design |
| Celik | 2022 | Effects of Occupational Therapy via Telerehabilitation on Occupational Balance, Well-Being, Intrinsic Motivation and Quality of Life in Syrian Refugee Children in COVID-19 Lockdown: A Randomized Controlled Trial | Children. 9: 485 | Population/ Intervention |
| Halty | 2022 | Support for Families During COVID‐19 in Spain: The iCygnus Online Tool for Parents | Child Psychiatry & Human Development. 53: 808–821 | Study design |
| Harper | 2021 | A homeschool‐based cognitive behavioral program to improve adolescent mental health | J Child Adolesc Psychiatr Nurs. 35: 179–188 | Study design |
| Haruna | 2020 | Perinatal mental health and COVID-19 in Japan | Psychiatry and Clinical Neurosciences. 74: 496–512 | Study design |
| Holzman | 2022 | Parenting in a Pandemic: Preliminary Support for Delivering Brief Behavioral Parent Training Through Telehealth | Behavior Modification 00(0): 1-26 | Study design |
| Hughes | 2022 | Implementing and Adapting the SAFETY Treatment for Suicidal Youth: The Incubator Model, Telehealth, and the Covid-19 Pandemic | Cognitive and Behavioral Practice. 29: 198–213 | Study design |
| Iachini | 2021 | Resources for Families during COVID-19: A Content Analysis of Information Provided on School District Web Sites | Children & Schools. 43(4): 209-2014 | Study design |
| Iacono | 2021 | Critical reflections and reflexivity on responding to the needs of LGBTQ+ youth in a global pandemic | Qualitative Social Work. 20(1–2): 479–486 | Study design |
| Ilari | 2022 | Perceptions of Parenting, Parent-Child Activities and Children’s Extracurricular Activities in Times of COVID-19 | Journal of Child and Family Studies. 31: 409–420 | Study design |
| Ineese-Nash | 2020 | Finding Our Power Together: Working with Indigenous Youth and Children during COVID-19 | Child & Youth Services. 41(3): 274-276 | Study design |
| Iordachescu | 2021 | Pregnancy during a pandemic: How stress affects pregnant women and the development of their babies during COVID-19 | Revista de psihologie. 67(2):153-168 | Study design |
| Iovino | 2021 | Teaching Simple Strategies to Foster Emotional Well-Being | Frontiers in Psychology. 12: 772260 | Study design |
| Jafarnejad | 2021 | The Effect of Education on Anxiety and Social Adjustment of Parents with Children with Corona Referred to Ali Asghar Hospital | International Journal of Early Childhood Special Education. 13(2): 932-939 | Population/ Intervention |
| Islam | 2021 | Mental Health of Children Amid COVID-19 Pandemic in Bangladesh: An Exploratory Observation | Asia Pacific Journal of Public Health. 33(4) 469–470 | Study design |
| Jafree | 2021 | Digital health literacy intervention to support maternal, child and family health in primary healthcare settings of Pakistan during the age of coronavirus: study protocol for a randomised controlled trial | BMJ Open. 11:e045163 | Population/ Intervention |
| Riegler | 2020 | Pilot Trial of a Telepsychotherapy Parenting Skills Intervention for Veteran Families: Implications for Managing Parenting Stress During COVID-19 | Journal of Psychotherapy Integration. 30(2): 290-303 | Study design |
| Jayesinghe | 2021 | The send stay and play summer initiative 2020 | Arch Dis Child 2021;106(Suppl 1):A1–A514 | Study design |
| Francis | 2020 | Tele-Wellness Supported App for Family Child Care Home Providers and Families to Promote Health, Family Engagement, and School Readiness Amid COVID-19 | NCT04453657  https://clinicaltrials.gov/ct2/show/NCT04453657 | Study design |
| Juarascio | 2021 | The Reward Re-Training protocol: A novel intervention approach designed to alter the reward imbalance contributing to binge eating during COVID-19 | Int J Eat Disord. 54: 1316–1322 | Population/ Intervention |
| Khan | 2021 | Treating Social Anxiety in an Era of Social Distancing: Adapting Exposure Therapy for Youth During COVID-19 | Cognitive and Behavioral Practice. 28: 669–678 | Study design |
| Behbahani | 2018 | Effects of Mindful Parenting Training on Clinical Symptoms in Children with Attention Deficit Hyperactivity Disorder and Parenting Stress: Randomized Controlled Trial | Iran J Med Sci. 43(6): 596-604 | COVID-19 |
| Layton | 2021 | Depression, Anxiety, and Mother-Infant Bonding in Women Seeking Treatment for Postpartum Depression Before and During the COVID-19 Pandemic | J Clin Psychiatry. 82(4): e1-6 | Study design |
| Lerthattasilp | 2021 | Effect of an online psychological support group on patients with COVID-19 in a Thai field hospital: a real world study | Journal of Health Research. 36(6) | Population/ Intervention |
| Lake, Limbix Health |  | A CBT-based mobile intervention as first line treatment for adolescent depressive symptoms during COVID-19 | NCT: 04524598  https://clinicaltrials.gov/ct2/show/NCT04524598 | Population/ Intervention |
| MacDonald | 2021 | Cohort profile: the Men and Parenting Pathways (MAPP) Study: a longitudinal Australian cohort study of men’s mental health and well-being at the normative age for first-time fatherhood | BMJ Open. 11: e047909. | COVID-19 |
| MacEvilly | 2020 | Adapting an emotional regulation and social communication skills group programme to teletherapy, in response to the COVID-19 pandemic | Irish Journal of Psychological Medicine. 39(4): 423-428 | Study design |
| MacKinnon | 2022 | Building Emotional Awareness and Mental Health (BEAM): A Pilot Randomized Controlled Trial of an App-Based Program for Mothers of Toddlers | Frontiers in Psychiatry. 13: 880972 | Population/ Intervention |
| Maguire-Jack | 2022 | Implementing Triple P during the COVID-19 pandemic with families at risk for substance use | Child Abuse & Neglect. 129: 105636 | Study design |
| Mahbub | 2020 | Humanistic and Mental Health Behaviorism in Shaping the Learning Process in Covid-19 Outbreak | International Journal of Pharmaceutical Research. 12(4): 3535-3539 | Study design |
| Siu | 2021 | Implementation Science and Impact Evaluation of PfR Programme: A hybrid cRCT design | NCT05105373  https://clinicaltrials.gov/ct2/show/NCT05105373 | COVID-19 |
| Malhotra | 2021 | Mindful Digital Program–based Interventions and their Role in Pregnancy and Fetal Outcomes | Journal of South Asian Federation of Obstetrics and Gynaecology. 13(3): 170-175 | Study design |
| Mann | 2021 | ICARE: Internet-based child-adult relationship enhancement for families at risk for emotional dysregulation and child maltreatment | Journal of the American Academy of Child & Adolescent Psychiatry. 60(10S): 75 | Study design |
| McDonough 2022 | 2022 | Effects of a remote, YouTube-delivered exercise intervention on young adults’ physical activity, sedentary behavior, and sleep during the COVID-19 pandemic: Randomized controlled trial | Journal of Sport and Health Science. 11: 145-156 | Population/ Intervention |
| Miller | 2021 | Health Effects of COVID-19 for Vulnerable Adolescents in a Randomized Controlled Trial | School Psychology. 36(5): 293–302 | COVID-19 |
| Mistree | 2021 | Instructional interventions for improving COVID-19 knowledge, attitudes, behaviors: Evidence from a large-scale RCT in India | Social Science & Medicine. 276: 113846 | Population/ Intervention |
| Monash University | 2021 | Evaluating the effects of an online parenting program with peer support (PiP-Plus) for parents of adolescents (aged 12 to 17) on parent confidence and parent and adolescent wellbeing during the COVID-19 pandemic | ACTRN12621000854831 | Study design |
| Newhouse | 2021 | “I’m fine.” “It’s fine.” “We are not fine!” – Providing behavioral health service to families in rural Appalachia during COVID-19 | Patient Education and Counseling. https://doi.org/10.1016/j.pec.2021.05.014 | Study design |
| Nicolaidou | 2021 | Building Primary-School Children’s Resilience through a Web-Based Interactive Learning Environment: Quasi-Experimental Pre-Post Study | JMIR Pediatr Parent. 4(2): e27958 | COVID-19 |
| Orgilés | 2020 | How Super Skills for Life may help children to cope with the COVID-19: Psychological impact and coping styles after the program | Revista de Psicología Clínica con Niños y Adolescentes. 7(3): 88-93 | Study design |
| Paliliunas | 2022 | Evaluating an ACT‐Based Brief Intervention for Educators Treatment Package on Reported Well‐Being and ACT‐Consistent Language in the Classroom | Behavior Analysis in Practice. https://doi.org/10.1007/s40617-022-00707-7 | Study design |
| Panteli | 2021 | Perceived Stress of Cypriot College Students During COVID-19 | European Journal of Psychology Open. 80(1–2): 31–39 | Population/ Intervention |
| Parker | 2021 | The Building Educators' Skills in Adolescent Mental Health Training Program for Secondary School Educators: Protocol for a Cluster Randomized Controlled Trial | JMIR Res Protoc. 10(2): e25870 | COVID-19 |
| Binda | 2022 | A Group Videoconferencing Intervention (C@nnected) to Improve Maternal Sensitivity: Protocol for a Randomized Feasibility Trial | JMIR Res Protoc. 11(8): e35881 | COVID-19 |
| Queen Mary University London | 2021 | A research study in Colombia, to build capacity in school mental health and to adapt an intervention called DIALOG+ for use in adolescents in post-conflict Colombia during the COVID-19 pandemic | ISRCTN14396374 | COVID-19 |
| Raccanello | 2020 | Development and Early Implementation of a Public Communication Campaign to Help Adults to Support Children and Adolescents to Cope With Coronavirus-Related Emotions: A Community Case Study | Frontiers in Psychology. 11: 2184 | Study design |
| Ramadhan | 2020 | Children’s Mental Health in the Time of COVID-19: How Things Stand and the Aftermath | Malays J Med Sci. 27(5): 196–201 | Study design |
| Rashid | 2020 | Real-Time WebRTC-Based Application for Psychological Support During COVID-19 | International Journal of Advanced Trends in Computer Science and Engineering. 9(4): 208-216 | Study design |
| Rauschenberg | 2021 | Social isolation, mental health, and use of digital interventions in youth during the COVID-19 pandemic: a nationally representative survey | Eur Psychiatry. 64(1): e20 | Study design |
| Ravindran | 2021 | Psychosocial Intervention Model of Kerala, India During Pandemic COVID-19: “Ottakkalla Oppamundu (You’re not alone, we’re with you)” | International Journal of Health Services. 51(4): 436–445 | Study design |
| Ren | 2021 | The Protective Roles of Exercise and Maintenance of Daily Living Routines for Chinese Adolescents During the COVID-19 Quarantine Period | Journal of Adolescent Health. https://doi.org/10.1016/j.jadohealth.2020.09.026 | Study design |
| Rhodes | 2020 | Experiences, Attitudes, and Needs of Users of a Pregnancy and Parenting App (Baby Buddy) During the COVID-19 Pandemic: Mixed Methods Study | JMIR Mhealth Uhealth. 8(12): e23157. | Study design |
| Ribeiro | 2021 | Telephone-based psychological crisis intervention: the Portuguese experience with COVID-19 | Counselling Psychology Quarterly. 34(3-4): 432-446 | Study design |
| Riegler | 2020 | Pilot Trial of a Telepsychotherapy Parenting Skills Intervention for Veteran Families: Implications for Managing Parenting Stress During COVID-19 | Journal of Psychotherapy Integration. 30(2): 290-303 | Study design |
| Roels | 2022 | Confident futures: Community-based organizations as first responders and agents of change in the face of the Covid-19 pandemic | Social Science & Medicine. 294:114639 | Study design |
| Romanowicz | 2021 | I-PCIT: Internet-delivered parent-child interaction therapy | Journal of the American Academy of Child & Adolescent Psychiatry. 60(10S): 75 | Study design |
| Sadeghi | 2021 | Supporting teen parents during COVID-19 | Platform Abstracts / Journal of Adolescent Health. 68: S20eS59 | Study design |
| Saju | 2020 | REaCH-Resiliency Engagement and Care in Health; A Telephone Befriending Intervention to Address The Psycho-Social Challenges of Vulnerable Population in The Context of COVID-19 Pandemic: An Exploratory Trial in India | Research Square. https://doi.org/10.21203/rs.3.rs-72843/v1 | Population/ Intervention |
| Saline | 2021 | Thriving in the New Normal: How COVID-19 has Affected Alternative Learners and Their Families and Implementing Effective, Creative Therapeutic Interventions | Smith College Studies in Social Work. 91(1): 1-28 | Study design |
| Sanz | 2020 | Consideraciones sobre conducta infantil y confinamiento en la crisis por COVID-19 desde la perspectiva funcional del Análisis de la conducta y ACT | International Journal of Psychology and Psychological Therapy. 20(2). 115-129 | Study design |
| Schwank | 2020 | Mental health of Urban Mothers (MUM) study: a multicentre randomized controlled trial, study protocol | BMJ Open. 10: e041133. | Outcome |
| Seddio | 2022 | Attention-deficit/hyperactivity disorder (ADHD) and anxiety during the COVID-19 pandemic: Implications for internalizing behaviors in college students | Journal of American College Health. https://doi.org/10.1080/07448481.2022.2069469 | Study design |
| Seegert | 2021 | Safety Education for Children Cannot Stop for a Pandemic: Transitioning an Injury Prevention Program to a Virtual Format | Early Childhood Education Journal. 49: 881–886 | Study design |
| Senol | 2021 | The effect of child neglect and abuse information studies on parents’ awareness levels during the COVID-19 pandemic | Children and Youth Services Review. 131: 106271 | Study design |
| Serlachius | 2021 | Pilot study of a well-being app to support New Zealand young people during the COVID-19 pandemic | Internet Interv. 26: 100464 | Study design |
| Serlachius | 2020 | Coping Skills Mobile App to Support the Emotional Well-Being of Young People During the COVID-19 Pandemic: Protocol for a Mixed Methods Study | JMIR Res Protoc. 9(10): e23716 | Population/ Intervention |
| Shahidi | 2020 | Physical activity during COVID-19 quarantine | Acta Paediatr. 109(10):2147-2148 | Study design |
| Shebab | 2021 | A Randomized Controlled Trial of Psychological Outcomes of Mobile Guided Resonant Frequency Breathing in Young Adults with Elevated Stress During the COVID-19 Pandemic | Dissertation, Graduate Faculty in Psychology, City University of New York | Population/ Intervention |
| Sheriff | 2021 | A Cultural Experience to support Mental Health in People Aged 16-24 During COVID-19 Compared to a Typical Museum Website: Study Protocol of an Online Randomised Controlled Trial | Research Square. https://doi.org/10.21203/rs.3.rs-181910/v1 | Population/ Intervention |
| Sobko | 2020 | Impact of outdoor nature‐related activities on gut microbiota, fecal serotonin, and perceived stress in preschool children: the Play&Grow randomized controlled trial | Scientific Reports. 10: 21993 | COVID-19 |
| Tarbox | 2021 | Taking ACTion: 18 Simple Strategies for Supporting Children With  Autism During the COVID-19 Pandemic | Behavior Analysis in Practice. 14: 1099–1127 | Study design |
| Bustamante-Ara | 2021 | Change Physical Activity and School Play Space. Radomized Study protocol. (Play&Cognition) | NCT05020041  https://clinicaltrials.gov/ct2/show/NCT05020041 | Outcome |
| Carson | 2022 | The Expansion of a Parent-focused Physical Literacy Intervention for Early Childhood Called PLAYshop | NCT05436197  https://clinicaltrials.gov/ct2/show/NCT05436197 | Outcome |
| Van Voorhees | 2020 | PATH 2 Purpose: Primary Care and Community-Based Prevention of Mental Disorders in Adolescents (P2P) | NCT04290754  https://clinicaltrials.gov/ct2/show/NCT04290754 | COVID-19 |
| Riggs | 2022 | Infant Mental Health Home Visiting Mitigates Impact of Maternal Adverse Childhood Experiences on Toddler Language Competence: A Randomized Controlled Trial. | J Dev Behav Pediatr. 43(4): e227-e236 | COVID-19 |
| Braungart-Rieker | 2017 | Efficacy of Family Programs for Improving Child and Family Health and Development | NCT03367845  https://clinicaltrials.gov/ct2/show/NCT03367845 | COVID-19 |
| Polanczyk | 2021 | Brief Internet-delivered Intervention for Children and Adolescents With Anxiety and Depression Symptoms | NCT05139433  https://clinicaltrials.gov/ct2/show/NCT05139433 | Population/ Intervention |
| Gualano | 2020 | Home-based Aerobic Training Among Adolescents With Chronic Diseases During COVID-19 Pandemic | NCT04458246  https://clinicaltrials.gov/ct2/show/NCT04458246 | Population/ Intervention |
| Vahabi | 2022 | Effects of Acceptance and Commitment Therapy (ACT) on Mental Health and Resiliency of Migrant Live-in Caregivers in Canada: Pilot Randomized Wait List Controlled Trial | JMIR Formative Research. 6(1): e32136 | COVID-19 |
| Van Lieshout | 2021 | Effect of Online 1-Day Cognitive Behavioral Therapy–Based Workshops Plus Usual Care vs Usual Care Alone for Postpartum Depression A Randomized Clinical Trial | JAMA Psychiatry. 78(11): 1200-1207 | Outcome |
| Kujawa | 2022 | Family Promoting Positive Emotions Pilot Study | NCT05223842  https://clinicaltrials.gov/ct2/show/NCT05223842 | Population/ Intervention |
| Markovic | 2020 | Effectiveness of Expressive Writing in the Reduction of Psychological Distress During the COVID-19 Pandemic: A Randomized Controlled Trial | Frontiers in Psychology. 11: 587282 | Population/ Intervention |
| Wang | 2021 | Predictors of mood, diabetes-specific and COVID-19-specific experiences among parents of early school-age children with type 1 diabetes during initial months of the COVID-19 pandemic | Pediatr Diabetes. 22: 1071–1080 | Population/ Intervention |
| Javanbakht | 2018 | Arts and Movement Therapies for Trauma | NCT03515564  https://clinicaltrials.gov/ct2/show/NCT03515564 | Study design |
| Weiss-Laxer | 2022 | Group Well-Child Care Model for Latino Children in Immigrant Families: Adapting to and Learning From the Coronavirus Disease 2019 (COVID-19) Context | Families, Systems, & Health. http://dx.doi.org/10.1037/fsh0000697 | Study design |
| Xie | 2022 | Building Emotional Awareness and Mental Health (BEAM): Study protocol for a phase III randomized controlled trial of the BEAM App-based program for mothers of children 18-36 months. | Trials. 23: 741 | Population/ Intervention |
| Rosenberg | 2021 | Implementing and Evaluating a Social-Emotional Learning Program for Refugee Children During the COVID-19 Pandemic | NCT04931888  https://clinicaltrials.gov/ct2/show/NCT04931888 | Study design |
| Prime | 2022 | Lausanne Trialogue Paradigm - Brief: A Family Model for Child Mental Health in a Community Setting (LTP-B) | NCT05356247  https://clinicaltrials.gov/ct2/show/NCT05356247 | Study design |
| Angrist, Young 1ove | 2021 | Empowering Girls: Health-seeking Behavior, Staying in School, and Preventing Risky Sex | NCT05022277  https://clinicaltrials.gov/ct2/show/NCT05022277 | COVID-19 |
| Yuan | 2021 | Mindfulness training on the resilience of adolescents under the COVID-19  epidemic: A latent growth curve analysis | Personality and Individual Differences. 172: 110560 | Study design |
| Zepeda | 2021 | iCOPE With COVID-19: A Brief Telemental Health Intervention  for Children and Adolescents During the COVID-19 Pandemic | Cognitive and Behavioral Practice. https://doi.org/10.1016/j.cbpra.2021.10.001 | Study design |

**Appendix C: Extracted data items**

First author(s)

Date published

Title

Country/region

Study source/journal

Study design

Participants

Eligibility criteria

Randomized patients

Included patients

Recruitment setting and procedure

Pandemic control measure(s) in place

Short description of intervention

Description of specific measure(s)

Setting of intervention

Mode of delivery

Digital component (+software name/producer)

Target group

Theory of intervention

Time of delivery

Duration/intensity/frequency

Provider of intervention

IASC broad category

IASC activity code(s)

Description of comparison

Mode of delivery of comparison

Time of delivery of comparison

Description of outcome

Effect measure/tool

Effect size

Mode/setting of data collection

Assessment time points

Total number of participants

Participants in intervention group

Outcome(s) in intervention group

Participants in comparison group

Outcome(s) in comparison group

Dropouts after baseline

Characteristics of groups similar

Allocation ratio

Statistical analyses

Intention-to-treat/per-protocol

Sample size (calculation)

Funding/financial sources

**Appendix D: Characteristics of included RCT and cRCT protocols**

**Table A2.** Characteristics of included RCT and cRCT protocols

| **First author** | **Year** | **Country** | **Study design** | **Study source** | **Sample size** | **Intervention** | **Mode of delivery** | **Outcomes*** | **Follow up** |
| --- | --- | --- | --- | --- | --- | --- | --- | --- | --- |
| **Chen** | 2020 | China | randomized delayed crossover open label controlled trial | Trials 2020:21:402 | 76 intended | Solution-focused brief therapy for adolescents | Online sessions | Anxiety, depression, coping | 6 weeks |
| **Park** | 2021 | South Korea | mixed-method randomized-controlled trial | ISRCTN  https://www.isrctn.com/ISRCTN42122907 | 22 final | Stimulated laughter therapy for mothers | Online group sessions | Depression, anxiety, parental stress | 2 weeks |
| **Eapen** | 2021 | Australia | RCT | BMC Health Services Research 2021:21:1240 | 70 intended, 22 final | Developmental surveillance platform to monitor child development and guide parents, link at-risk individuals to local support structures | Digital information, phone contact with providers, in-person meetings | Parent mental health, parent psychosocial needs, developmental risk | >1 year |
| **Ehrenreich** | 2020 | USA | RCT | ClinicalTrials.gov  NCT04431856 | 80 actual | Cognitive and behavioural strategies for parents to manage own distress, anxiety, sadness and strategies related to parenting | N.i. | Anxiety, depression, PTSD, parenting overprotection/overcontrol, parenting emotional warmth, parent accommodation for child anxiety | 12 weeks |
| **Fitzpatrick** | 2021 | USA | RCT | ClinicalTrials.gov  NCT04806321 | 522 actual | Single session intervention on problem-solving skills, improving mental health and wellbeing for adolescents | Digital, self-guided | Anxiety, depression, hopefulness, hopelessness, misbehavior | 1 year |
| **Miklosi** | 2020 | Hungary | RCT | ISRCTN  ISRCTN15254871 | 300 intended | Parent training to reduce parental and children´s stress | Digital, self-guided | Perceived stress, psychological wellbeing, parenting stress, parenting behavior, quality of life | 3 months |
| **Huang** | 2020 | China | RCT | ChiCTR  ChiCTR2000033433 | 300 intended | Internet-based cognitive behavior intervention for perinatal depression | Digital, self-guided and written feedback to provider | Anxiety, depressive symptoms, parenting stress, partner´s support | 1 year |
| **Kostyrka-Allchorne** | 2021 | United Kingdom | RCT | Trials 2021; 22.267 | 616 intended | Parent support for managing children´s behaviour, reduce conduct problems and family conflict | Digital, self-guided and parent-to-parent communication platform | Parent-reported child conduct problems | 2 months^#^ |
| **Steinbauer** | 2021 | Austria | RCT | ClinicalTrials.gov  NCT04724616 | 60 estimated | Interdisciplinary psychoeducational intervention program (e.g. understanding containment measures) for children | Online group sessions | Change of emotional outcome | 3 months |
| **Ratcliff** | 2021 | USA | RCT | ClinicalTrials.gov  NCT04980365 | 105 anticipated | Mindfulness-/gratefulness-based personal education, enrichment and stress reduction for parents | App-based, self-guided | Parental stress, mindfulness, gratitude, positive and negative affect, child-parent relationship, perceived stress, perception of intimate relationship status | 6 weeks |
| **Skeen** | 2020 | Zambia, Tanzania, Uganda | RCT | ISRCTN  ISRCTN77689525 | 734 final | Intervention to promote child social, emotional and language development, responsive parenting and parental mental wellbeing | Digital, self-guided and group chat sessions | Social and emotional outcomes, parenting behaviors, parenting stress, parental mental health | 6 weeks |
| **Yilmaz** | 2021 | Turkey | RCT | CliicalTrials.gov  NCT04815057 | 176 actual | Education on wellness for children | N.i. | Quality of life | 3 months |
| **Roos** | 2021 | Canada | RCT | ClinicalTrials.gov  NCT04925258 | 40 estimated | Psychoeducational (emotion-focused) parenting videos, structured family activities, parenting support group | Digital, self-guided, online group sessions | Parenting stress, child mental health and behavior, emotion-focused parenting practices, mindful parenting practices, parental/caregiver resilience | 3 months |
| **Díaz Zavala** | 2021 | Mexico | RCT pilot | Rev Esp Nutr Hum Diet. 2021; 25 (Supl. 2): e1320. doi: 10.14306/renhyd.25.S2.1320  ClinicalTrials.gov  NCT04772859 | 54 actual | Nutrition education for children | Online group sessions | Quality of life | 4 months |
| **Taylor** | 2020 | United Kingdom | RCT | ISRCTN  ISRCTN12890382 | 444 total final enrolled | Therapist-supported, parent-led cognitive behavioural therapy | Telephone sessions, online platform | Anxiety, comorbid emotional and behavioral problems, quality of life | 26 weeks |
| **Thulasi** | 2021 | India | RCT | CTRI  CTRI/2021/02/031543 | 500 intended | Yoga sessions for children | Online (group session) | Mental health, health-related quality of life, wellbeing | 12 weeks |

^#^ presumed follow up

**Appendix E: Funding sources of included studies**

**Table A3.** Funding sources of included studies

| **Study** | **Funding/financial resources** |
| --- | --- |
| **Altuntas 2022** | No specific funding mentioned |
| **Chen 2021** | The project of philosophy and social science planning in Zhejiang Province (19NDJC141YB) |
| **Ding 2020** | National social sciences Fund of China (19BTY017) |
| **Karadag 2021** | No specific funding mentioned |
| **Malboeuf-Hurtubise (art) 2021** | Fonds de Recherche du Québec Société et Culture to CMH |
| **Malboeuf-Hurtubise (philosophy) 2021** | Bishop’s University support to new faculty fund  Social Sciences and Humanities Research Council of Canada to CMH |
| **Pavarini 2021** | University of Oxford’s COVID-19: Economic, Social, Cultural, & Environmental Impacts – Urgent Response Fund  Wellcome Trust [104825/Z/14/Z and 203132/Z/16/Z]  NIHR Oxford Health Biomedical Research Centre [IS-BRC-1215–20005] |
| **Schleider 2022** | Office of the Director, National Institutes of Health under an “Emergency COVID-19 Competitive Revision Award” (grant DP5OD028123) |
| **Shao 2021** | No specific funding mentioned |
| **Tymofieva 2022** | UCSF Research Resident Training Program R25 grant Weill Institute for Neurosciences Weill Award for Junior Investigators in the Neurosciences Impacted by COVID-19 Setbacks  National Center for Complementary and Integrative Health (NCCIH) R21AT009173  National Center for Advancing Translational Sciences (CTSI) and the National Institutes of Health, through UCSF-CTSI UL1TR001872  American Foundation for Suicide Prevention (AFSP) SRG-1-141-18  Swedish Research Council 350-2012-303  National Institute of Mental Health (NIMH) R01MH085734  Brain and Behavior Research Foundation (formerly NARSAD) |
| **Xu 2021** | The National Social Science Fund of China (19BTY017) |
| **Zhang 2021** | 2020 Key Research & Development Plan Project of Shandong Province (soft science project: 2020RKB01317) |
| **Zheng 2021** | Construction Project of High-Level Hospitals in Guangdong Province (303020107, 303010303058) National Natural Science Foundation of China (81530028, 81721003)  Clinical Innovation Research Program of Guangzhou Regenerative Medicine and Health Guangdong Laboratory (2018GZR0201001)  research units of Ocular Development and Regeneration, Chinese Academy of Medical Sciences (2019-I2M-5-005)  Local Innovative and Research Teams Project of the Guangdong Pearl River Talents Program (2017BT01S138)  State Key Laboratory of Ophthalmology, Zhongshan Ophthalmic Center, Sun Yat-sen University |

**Appendix F: Risk of bias summary and graph**


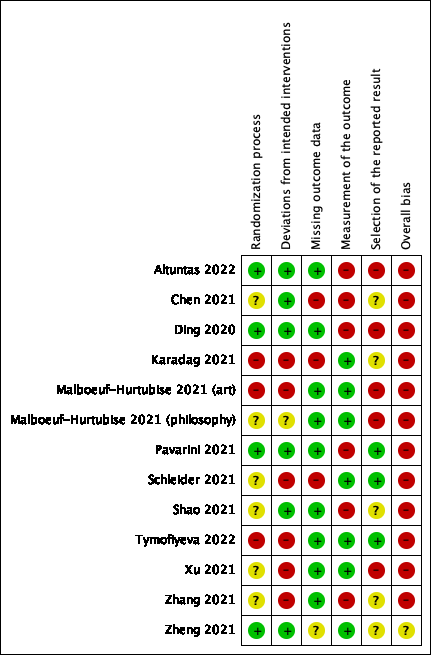


**Figure A1.** Risk of bias summary


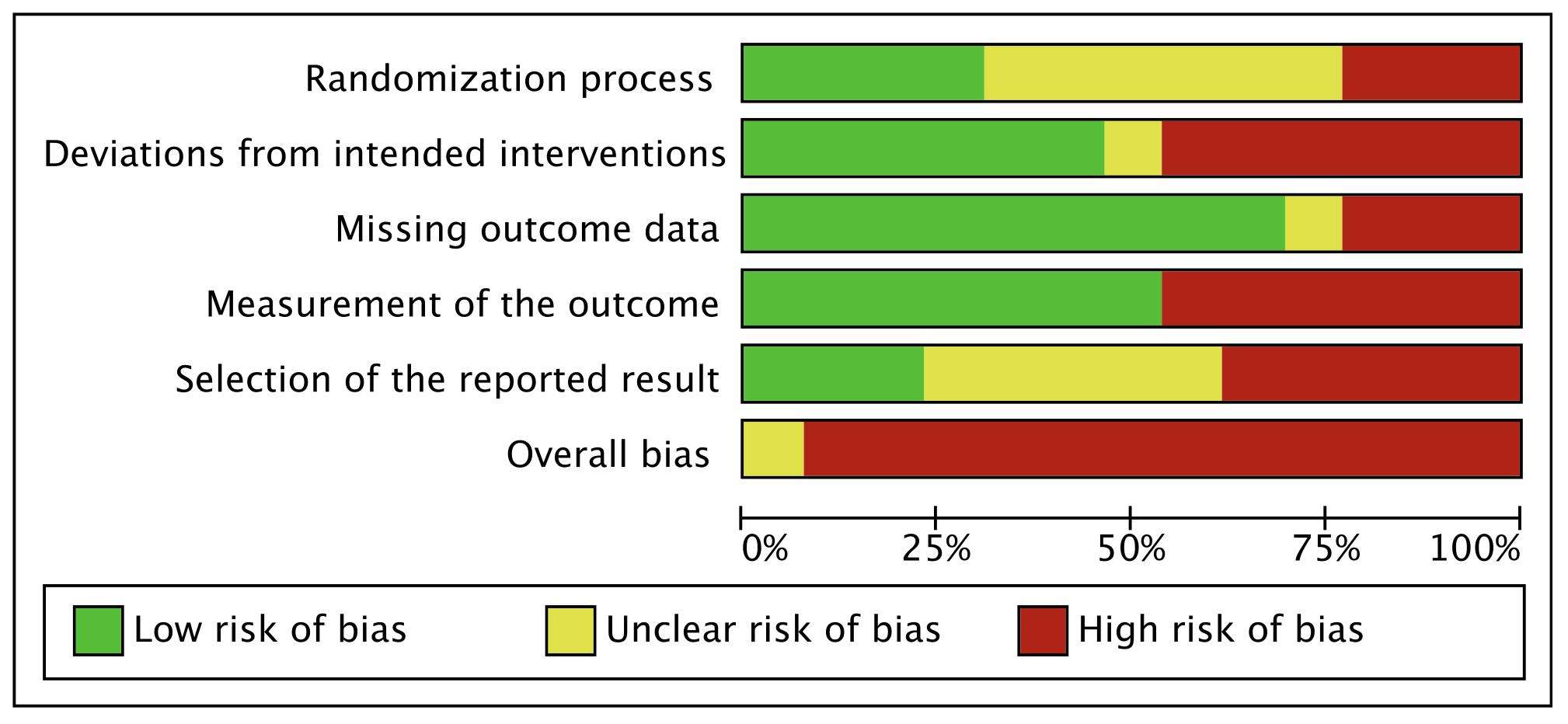


**Figure A2.** Risk of bias graph

**Appendix G: Funnel plots**


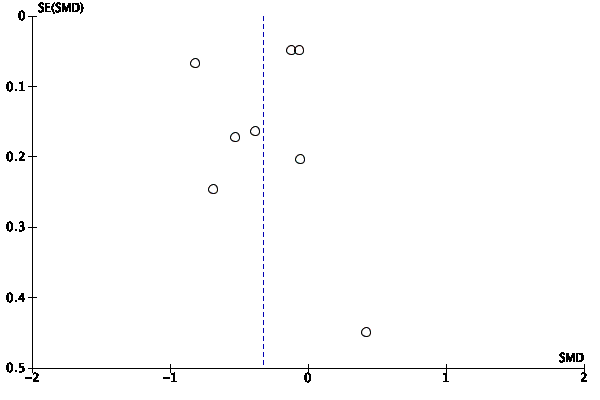


**Figure A3.** Funnel plot, outcome: anxiety


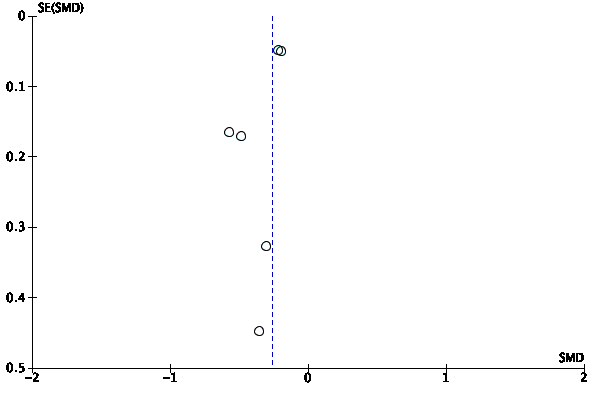


**Figure A4.** Funnel plot, outcome: depressive symptoms

**Appendix H: Combined effects of various interventions on sleep disturbance**


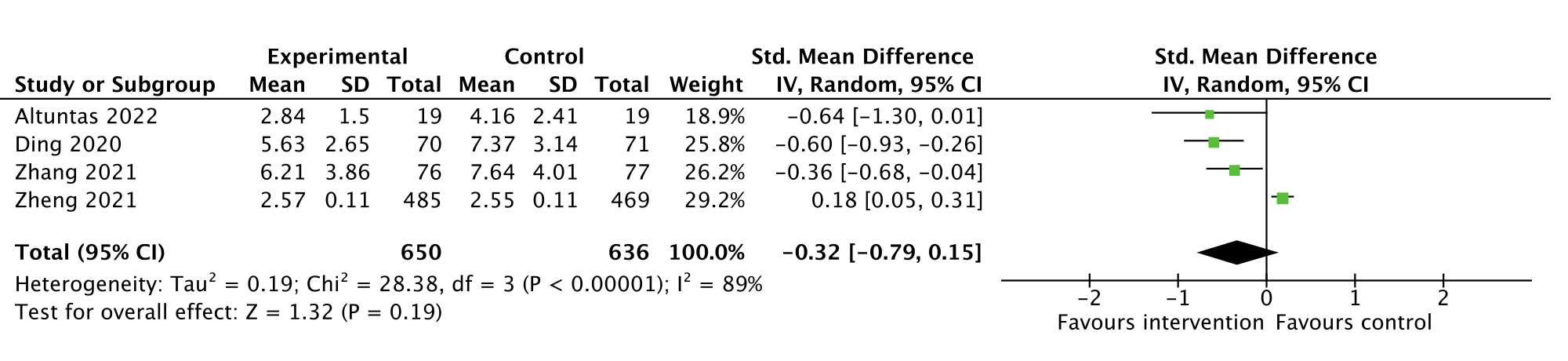


**Figure A5.** Combined effects (standardized mean difference) of various psychosocial interventions on sleep disturbance among children and adolescents (forest plot)

**Appendix I: Subgroup analyses**

**Table A4.** Subgroup analyses for the outcomes anxiety and depressive symptoms

|  | **All** | **Length and intensity of the intervention** | | **Integration of a physical activity component** | **Activity codes** | | | **Personal**  **interactions** | **Pre-selected**  **study participants** |
| --- | --- | --- | --- | --- | --- | --- | --- | --- | --- |
|  |  | **At least twice/week, duration 7-12 weeks** | Once/week or less frequent, duration 1-5 weeks |  | Activity code 5 | Activity code 8 | Activity code 3 |  |  |
| **Anxiety** | -0.32  (-0.60—0.05) | -0.50  (-0.71, -0.29) | -0.20  (-0.58, 0.17) | -0.65  (-0.95, -0.35) | -0.53  (-0.82, -0.25) | -0.18  (-0.35, -0.00) | -0.53  (-0.82, -0.25) | -0.53  (-0.82, -0.25) | -0.50  (-0.71, 0.29) |
|  | N=8, I^2^:92% | N=3, I^2^:0% | N=5, I^2^:95% | N=3, I^2^:67% | N=5, I^2^:71% | N=5, I^2^:61% | N=5, I^2^:72% | N=6, I^2^:71% | N=3, I^2^:0% |
| **Depressive symptoms** | -0.27  (-0.38—0.16) | -0.50  (-0.72, -0.28) | -0.21  (-0.29, -0.13) | -0.51  (-0.80, -0.23) | -0.52  (-0.74, -0.29) | -0.25  (-0.37, -0.13) | -0.49  (-0.71, -0.28) | -0.49  (-0.71, -0.28) | - |
|  | N=6, I^2^:26% | N=3, I^2^=0% | N=3, I^2^:0% | N=2, I^2^=0% | N=3, I2=0% | N=4, I^2^=37% | N=4, I^2^:0% | N=4, I^2^:0% | - |

Activity code 3: Strengthening community and family support

Activity code 5: Psychological support in education.

Activity code 8: Psychological intervention. n: number of interventions included in meta-analysis

I^2^: indicator for heterogeneity
